# Exploring the Complex Relationship between Gut Microbiota and Risk of Colorectal Neoplasia Using Bidirectional Mendelian Randomization Analysis

**DOI:** 10.1158/1055-9965.EPI-22-0724

**Published:** 2023-04-03

**Authors:** Wanxin Li, Xuan Zhou, Shuai Yuan, Lijuan Wang, Lili Yu, Jing Sun, Jie Chen, Qian Xiao, Zhongxiao Wan, Ju-Sheng Zheng, Cai-Xia Zhang, Susanna C. Larsson, Susan M. Farrington, Philip Law, Richard S. Houlston, Ian Tomlinson, Ke-Feng Ding, Malcolm G. Dunlop, Evropi Theodoratou, Xue Li

**Affiliations:** 1 Department of Big Data in Health Science School of Public Health, Centre of Clinical Big Data and Analytics of The Second Affiliated Hospital, Zhejiang University School of Medicine, Hangzhou, China.; 2 Unit of Cardiovascular and Nutritional Epidemiology, Institute of Environmental Medicine, Karolinska Institutet, Stockholm, Sweden.; 3 Colorectal Surgery and Oncology, Key Laboratory of Cancer Prevention and Intervention, Ministry of Education, The Second Affiliated Hospital, Zhejiang University School of Medicine, Hangzhou, China.; 4 Department of Nutrition and Food Hygiene, School of Public Health, Soochow University, Suzhou, China.; 5 Key Laboratory of Growth Regulation and Translational Research of Zhejiang Province, School of Life Sciences, Westlake University, Hangzhou, China.; 6 Department of Epidemiology, School of Public Health, Sun Yat-sen University, Guangzhou, China.; 7 Unit of Medical Epidemiology, Department of Surgical Sciences, Uppsala University, Uppsala, Sweden.; 8 Colon Cancer Genetics Group, Institute of Genetics and Cancer, University of Edinburgh, Edinburgh, United Kingdom.; 9 Cancer Research UK Edinburgh Cancer Research Centre, Institute of Genetics and Cancer, University of Edinburgh, Edinburgh, United Kingdom.; 10 Division of Genetics and Epidemiology, The Institute of Cancer Research, London, United Kingdom.; 11 MRC Human Genetics Unit, Institute of Genetics and Cancer, University of Edinburgh, Edinburgh, United Kingdom.; 12 Centre for Global Health, Usher Institute, University of Edinburgh, Edinburgh, United Kingdom.; 13 The Key Laboratory of Intelligent Preventive Medicine of Zhejiang Province, Hangzhou, China.

## Abstract

**Background:**

Human gut microbiome has complex relationships with the host, contributing to metabolism, immunity, and carcinogenesis.

**Methods:**

Summary-level data for gut microbiota and metabolites were obtained from MiBioGen, FINRISK and human metabolome consortia. Summary-level data for colorectal cancer were derived from a genome-wide association study meta-analysis. In forward Mendelian randomization (MR), we employed genetic instrumental variables (IV) for 24 gut microbiota taxa and six bacterial metabolites to examine their causal relationship with colorectal cancer. We also used a lenient threshold for nine apriori gut microbiota taxa as secondary analyses. In reverse MR, we explored association between genetic liability to colorectal neoplasia and abundance of microbiota studied above using 95, 19, and 7 IVs for colorectal cancer, adenoma, and polyps, respectively.

**Results:**

Forward MR did not find evidence indicating causal relationship between any of the gut microbiota taxa or six bacterial metabolites tested and colorectal cancer risk. However, reverse MR supported genetic liability to colorectal adenomas was causally related with increased abundance of two taxa: *Gammaproteobacteria* (*β* = 0.027, which represents a 0.027 increase in log-transformed relative abundance values of *Gammaproteobacteria* for per one-unit increase in log OR of adenoma risk; *P* = 7.06×10^−8^), *Enterobacteriaceae* (*β* = 0.023, *P* = 1.29×10^−5^).

**Conclusions:**

We find genetic liability to colorectal neoplasia may be associated with abundance of certain microbiota taxa. It is more likely that subset of colorectal cancer genetic liability variants changes gut biology by influencing both gut microbiota and colorectal cancer risk.

**Impact:**

This study highlights the need of future complementary studies to explore causal mechanisms linking both host genetic variation with gut microbiome and colorectal cancer susceptibility.

## Introduction

Colorectal cancer is globally recognized as the third most prevalent cancer and ranks as the fourth major contributor to cancer-related mortality (1, 2). The colon is the most heavily colonized part of the gastrointestinal tract (3) by microbiota, which is notable given that it is also the region of the gastrointestinal most commonly affected by cancer in economically developed countries (1).

The human gut microbiome has a complex relationship with the host, contributing to the absorption of nutrients, metabolism, immunity, and carcinogenesis (4, 5). Human gut microbiota in healthy individuals consists primarily of two predominant anaerobic phyla: *Firmicutes* and *Bacteroidetes.* However, their proportions and associated species are in a dynamic state and change over time, even within the same individual (6). Several studies have proposed a potential causal relationship between changes in the gut microbiome and the development of colorectal cancer, possibly via chronic metabolic and inflammatory changes (7–9). Meanwhile, gut microbial metabolites such as trimethylamine N-oxide (TMAO) have been also found to likely promote persistent inflammation and weaken host immunity (10), which might increase the risk of colorectal cancer.

Observational studies have linked multiple fecal microbiota (i.e., *Enterococcus, Porphyromonas, Salmonella, Pseudomonas, Peptostreptococcus, Actinomyces, Fusobacterium, Bifidobacterium*, and *Roseburia*) to colorectal neoplasia (11). The former seven taxa are consistently reported to be enriched in patients with colorectal cancer compared with healthy individuals, whilst the latter two consistently reported to be more abundant in colorectal cancer–free individuals (11). However, observational studies are unlikely to infer a causal relationship given that the studies conducted to date have been cross-sectional or case–control in design, and so are prone to confounding, reverse causality, and bias.

Mendelian randomization (MR) is an analytic approach which employs germline genetic variants as instrumental variables (IV) for exposures (12). The genetic variants, being randomly distributed during conception, are immune to reverse causality and confounding factors, thus providing estimates of disease risk with minimal interference from extraneous variables. By doing so, MR overcomes the limitations of conventional epidemiologic studies and produces reliable results, provided that pleiotropy (i.e., the phenomenon where genetic variants affect disease outcomes through alternative pathways) is absent (12). Here, we have conducted two-sample MR analyses to determine the causal relationship of gut microbiota and their metabolites with colorectal neoplasia. Using a reverse MR approach, we also tested whether SNPs associated with colorectal cancer and with adenomas are causally associated with specific microbiota to test whether tumor propensity and/or presence influences the gut microbiota.

## Materials and Methods

### Study design

**Figure 1.**
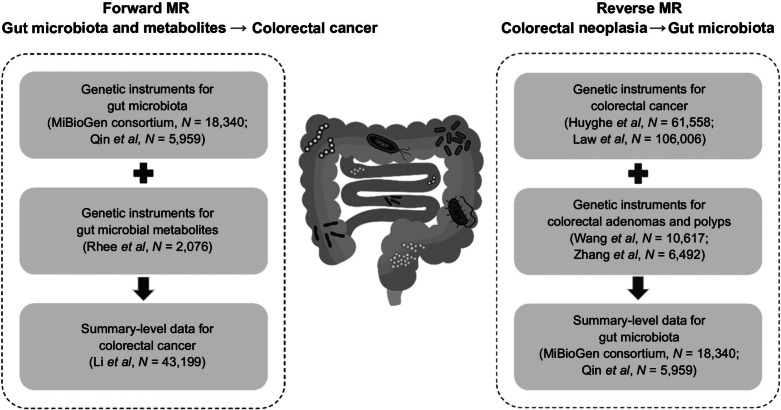
Study design. Schematic representation of the two-sample bi-directional MR analysis. MR was used to evaluate the causal relationships between gut microbiome as well as metabolites and colorectal neoplasia. Three key assumptions of MR: (1) genetic variants must be associated with exposures; (2) there should be no confounders between the genetic variants and the outcomes; (3) genetic variants must affect outcomes only through exposures, not through other pathways.

We adopted a bidirectional two-sample MR strategy to evaluate the causal relationship between gut microbiota and risk of colorectal neoplasia. The forward MR analysis was designed to investigate the causal effects of gut microbiota and related metabolites on colorectal cancer risk, while the reverse MR to examine whether the genetic liability to colorectal neoplasia (i.e., colorectal cancer, adenomas, and polyps) influenced the abundance of the gut microbiota. The study design is shown in Fig. 1. Details of the data sources are summarized in Table 1.

**Table 1.** Detailed information on data sources.

Exposure or outcome	Participants included in analysis	Adjustments	Number of genetic instruments			PubMed ID and/or URL
						
Gut microbiota taxa	18,340 European-descent individuals	Age and any necessary study-specific covariates	24 gut microbiota taxa	*P* < 5 × 10^−8^	27	33462485 https://mibiogen.gcc.rug.nl/
			*Gammaproteobacteria*	*P* < 1 × 10^−5^	9	
			*Lactobacillales*		19	
			*Enterobacteriaceae*		11	
			*Porphyromonadaceae*		10	
			*Actinomyces*		10	
			*Bifidobacterium*		18	
			*Roseburia*		18	
			*Peptostreptococcaceae*		16	
	5,959 European-descent individuals	Age, sex, genotyping batch and the top ten genetic principal components	*Fusobacteriaceae*		28	35115689
Gut microbial metabolites	2,076 European-descent individuals	Age, sex	Choline	6		23823483
			Betaine	13		
			Carnitine	12		
			TMAO	8		
			GABA	10		
			Propionic acid	2		
Colorectal cancer^a^	16,871 colorectal cancer cases and 26,328 controls of European ancestry	Age, sex and any necessary study-specific covariates	—			32638365
Colorectal cancer^b^	23,262 colorectal cancer cases and 38,296 controls of European ancestry	Age, sex and any necessary study-specific covariates	52			30510241
	34,627 Colorectal cancer cases and 71,379 controls of European ancestry	Age, sex and any necessary study-specific covariates	43			31089142
Colorectal adenomas	Discovery phase: 139 advanced adenoma cases and 1,267 controls of European ancestry; Validation phase: 4,175 colorectal adenoma cases and 5,036 controls of European ancestry	Age, sex, family history, prior aspirin use, and treatment.	19			24084763
Colorectal polyps	2,473 cases (1,831 with adenomas and 642 with hyperplastic polyps only) and 4,019 controls of European ancestry	Age, sex, body mass index, cigarette smoking and alcohol drinking.	7			23027627

^a^Summary-level data for colorectal cancer.

^b^Genetic instruments for colorectal cancer were extracted from two different GWAS studies. For replicated SNPs, the ones from GWAS with larger case number were chosen.

### Data sources for gut microbiome and metabolites

Summary statistics of the genetic contributions to gut microbiota taxa were obtained from the MiBioGen consortium (13) and FINRISK study (14). As we know, the MiBioGen consortium is the most extensive attempt to scrutinize the correlations between host-genetics and microbiome on a populace level, encompassing 18,340 participants from 24 European collectives, and exhibiting a vast geographic reach, in conjunction with a substantial sample size. Details on the MiBioGen consortium, including geographic regions, recruitment processes and other characteristics have been described in the published genome-wide association study (GWAS) previously (13). Each cohort obtained ethical approval and consent to participate in accordance with the local regulations and institutional requirements. The FINRISK 2002 cohort (FR02) study comprised of 5,959 European individuals and has been previously described in detail (14). The FINRISK population surveys, which aim to track the prevalence of cardiovascular and other noncommunicable disease risk factors among the Finnish population, have been conducted at 5-year intervals since 1972. The current investigation uses data from the FR02 study, which included men and women between the ages of 25 and 74 years from six distinct geographic regions in Finland. The sample was stratified by sex, region, and 10-year age group, with each stratum consisting of 250 participants. The research protocol for FR02 was approved by the Coordinating Ethical Committee of the Helsinki and Uusimaa Hospital District (Ref. 558/E3/2001), and all participants provided informed consent. The study was conducted in compliance with the ethical principles outlined in the World Medical Association's Declaration of Helsinki.

To complement with gut microbiome-derived metabolites, we selected six plasma metabolites with available GWAS data for analyses. Genetic predictors for choline, betaine, carnitine, TMAO, gamma-aminobutyric acid (GABA), and propionic acid were derived from a GWAS on human metabolome (15), which included 2,076 Europeans from the Framingham Heart Study (FHS) Offspring Cohort. The family-based FHS Offspring Cohort involved 2,076 participants belonging to 873 sibships, who underwent metabolic profiling and genome-wide genotyping. The study protocol was granted ethical clearance by the Boston University Medical Center Institutional Review Board, and all participants provided their informed consent.

### Selection of genetic instruments for MR analyses

For the forward MR analyses, SNPs associated with gut microbiota within the MiBioGen consortium at the GWAS significance level (*P* < 5×10^−8^) were selected as IVs. In total, 211 taxa (131 genera, 35 families, 20 orders, 16 classes, and 9 phyla) were eligible for the mbQTL (microbial quantitative trait locus) mapping analysis and of these 24 gut microbiota taxa met the *P* value threshold of 5×10^−8^ which were included in our forward MR analysis. On the basis of our previous systematic review of epidemiologic observational studies, nine gut microbiota taxa (i.e., *Enterococcus, Porphyromonas, Salmonella, Pseudomonas, Peptostreptococcus, Actinomyces, Fusobacterium, Bifidobacterium*, and *Roseburia*) were found to be consistently associated with the risk of colorectal neoplasia (11). Given that there were only three available SNPs (i.e., rs7322849, rs998451 for genus *Bifidobacterium* and rs61841503 for genus *Peptostreptococcaceae*) using the strict 5×10^−8^ for the nine gut microbiota taxa of interest which present with supporting evidence in their associations with colorectal neoplasm, we chose to use a lenient *P* value threshold of 1×10^−5^ as indicated by the MiBioGen as secondary analyses. Overall, we obtained SNPs associated with the abundance of the family *Fusobacteriaceae* from FR02 cohort (14) and SNPs associated with the other eight gut microbiota taxa at the genus or upper level (i.e., family, order, class) from the MiBioGen consortium (13). Our selection process aligns with a previous study which demonstrated that microbial features exhibit the greatest explained variance with associated SNPs falling below a significance threshold of *P* < 1×10^−5^ (16). To avoid issues with co-linearity between SNPs and each trait, we removed SNPs that exhibited linkage disequilibrium (LD, *r*^2^ > 0.01) and only retained SNPs with the smallest *P* values for their respective traits. After LD pruning, 27 SNPs associated with 24 gut microbiota taxa (at a stringent *P* value threshold of 5×10^−8^) and nine, 19, 11, 10, 28, 10, 18, 18, and 16 genetic IVs were included to proxy the abundance of *Gammaproteobacteria*, *Lactobacillales*, *Enterobacteriaceae*, *Porphyromonadaceae*, *Fusobacteriaceae*, *Actinomyces*, *Bifidobacterium*, *Roseburia*, and *Peptostreptococcaceae* (at a lenient *P* value threshold of 1×10^−5^; Supplementary Table S1–2). Similarly, 6, 13, 12, 8, 10, and 2 IVs were selected as proxies for plasma choline, betaine, carnitine, TMAO, GABA, and propionic acid, respectively (Supplementary Table S3). Regarding the reverse MR analysis, because there is no open available summary data for metabolites, we only used summary-level data of the microbiota taxa from the MiBioGen consortium and the FR02 cohort (13, 14).

### Data sources for colorectal neoplasia

Summary-level data on colorectal cancer used in forward MR analyses were obtained from our previous meta-analysis of 12 GWASs, namely CCRR1, CCFR2, COIN, CORSA, Croatia, DACHS, FIN, NSCCG-OncoArray, SCOT, UK1, and VQ58, which comprise 16,871 cases and 26,328 controls (17). Each dataset underwent application of standard quality control measures. For reverse MR, to increase the proportion of variance explained, we extracted all SNPs associated with colorectal cancer at the genome-wide significance level (*P *< 5 × 10^−8^) from two latest and largest colorectal cancer GWASs. Huyghe and colleagues performed a genome-wide association analysis of 23,262 cases and 38,296 controls (18), and Law and colleagues reported a genome-wide association analysis of 34,627 colorectal cancer cases and 71,379 controls of European ancestry (19). We used this comprehensive list of SNPs for colorectal cancer but only adopted the estimates from study with larger sample sizes (i.e., Law and colleagues; ref. 19). After excluding SNPs in LD, 95 SNPs were used as IVs for colorectal cancer in the reverse MR analyses (Supplementary **Table S4**). SNPs associated with colorectal adenomas were selected from a GWAS including 1,406 Caucasian individuals participating in the Adenoma Prevention with Celecoxib trial, 4,175 cases of familial colorectal adenoma and 5,036 control subjects of European ancestry (20). SNPs associated with polyps were selected from the Tennessee Colorectal Polyp Study, which included 2,473 cases and 4,019 controls (21). After removing those in LD, we obtained 19 SNPs for colorectal adenomas and 7 SNPs for colorectal polyps as IVs (Supplementary **Table S5**).

### Statistical analysis

We excluded SNPs with missing values and without proxy SNPs (*r*² > 0.8) in the outcome data source. We endeavored to deduce positive strand alleles by using allele frequencies for palindromes, with the default and conservative approaches and summary statistics were harmonized using the effect allele frequency such that all outcome effect estimates reflected the exposure-increasing effect allele. For each genetic instrument, β_GP_ represents the estimated association between the genetic variant and the exposure, while β_GD_ represents the estimated association between the same genetic variant and the outcome. By using these estimates, the causal estimates can be derived using the Wald ratio formula (β_GD_/β_GP_). Consequently, the estimates in the forward MR should be interpreted as changes in colorectal cancer risk for per one-unit increase in log-transformed relative abundance values of gut microbiota and for per one-SD increase in plasma metabolites levels, while the estimates in the reverse MR should be interpreted as changes in the abundance of gut microbiota for per one-unit increase in the log OR of colorectal neoplasia. For microbiota taxa with only one SNP, we employed the Wald ratio method for the MR analysis. For exposures with at least 3 IVs, MR estimates were computed using the inverse variance weighted random-effects (IVW-RE) model and complemented by five sensitivity analyses, namely the weighted median (22), weighted mode (23), MR-Egger regression (24), MR-PRESSO (25), and leave-one-out analysis. When applying the weighted median method, it is essential that at least 50% of the weight is derived from valid IVs to ensure consistency (22). In cases where the largest cluster SNPs are valid, the weight mode method can provide an unbiased causal effect estimate (23). MR-Egger correction method can be employed to address directional horizontal pleiotropy with decreased statistical power (24). MR-PRESSO can detect and remove potential outliers among IVs and provide causal estimates after the removal of the identified outliers (25). The leave-one-out analysis can be used to assess whether the association is driven by a single SNP. The heterogeneity among estimates of genetic instruments can be evaluated using Cochran's Q value and the *P* value for intercept in MR-Egger can be used to detect directional horizontal pleiotropy (24). The strength of instruments was evaluated by the *F*-statistic (Supplementary Methods) and the *F*-statistic being less than 10 implied weak instrument bias (26). To address the issue of multiple testing correction, FDR was employed. 39 and 124 independent tests were considered in the forward and reverse MR analyses, respectively. FDR < 0.05 was used as the threshold to indicate the strength of evidence against the null hypothesis. All analyses were performed using the TwoSampleMR (23) and MR-PRESSO (25) R packages in R software 4.1.1.

### Ethics approval

Because it relied solely on summary statistics from published GWAS and no utilization of individual-level data, this study did not require ethical approval.

### Data availability

All data examined in this research is comprehensively presented in this published article and its supplementary materials.

## Results

### Effects of gut microbiota taxa and related metabolites on colorectal cancer risk

In the forward MR analyses, we observed little associations between the abundance of gut microbiota taxa and colorectal cancer risk in the Wald ratio, IVW-RE method or in any of the sensitivity analysis (Supplementary Tables S6–S7). Moderate-to-high heterogeneity was observed in the association between abundance of *Roseburia* and colorectal cancer risk (*P* for heterogeneity = 0.001), and suggestive pleiotropy was observed in the MR analysis of *Lactobacillales* (*P* for pleiotropy = 0.017). For the analyses on gut microbiota taxa, there was a very low risk of weak instruments bias, given the *F*-statistics of all the genetic IVs were above 10. No association between the six gut microbial metabolites and colorectal cancer risk was observed in either the main IVW-RE or in any of the sensitivity analysis (Supplementary Table S8).

### Effects of liability to colorectal neoplasia on gut microbiota

In the reverse MR analyses, we found some evidence suggesting that genetic liability to colorectal neoplasia was associated with the abundance of gut microbiota.

For per one-unit increase in the log OR of genetic liability to colorectal cancer, the abundance of *Tyzzerella3* would have 0.066 [95% confidence intervals (CI), −0.123–0.009; *P* = 0.024] lower log-transformed relative abundance, based on the IVW-RE model. Although it did not survive the multiple test correction, the result was consistent across all the sensitivity analyses.

**Figure 2.**
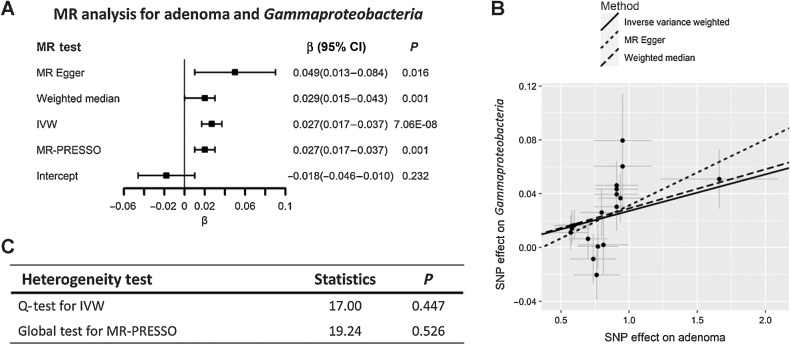
Effects of genetic liability to adenoma on *Gammaproteobacteria* from MiBioGen consortium. **A** and **C,** Forest plot for summarizing the results of all MR methods and heterogeneity tests of IVW and MR-PRESSO. **B**, Scatter plot for comparison of MR methods. Abbreviations: Q-test, Cochran's Q statistic heterogeneity test. MR-PRESSO, MR pleiotropy residual sum and outlier test.

**Figure 3.**
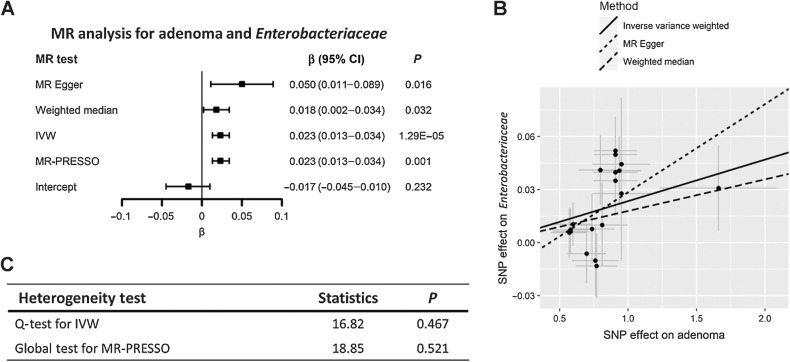
Effects of genetic liability to adenoma on *Enterobacteriaceae* from MiBioGen consortium. **A** and **C,** Forest plot for summarizing the results of all MR methods and heterogeneity tests of IVW and MR-PRESSO. **B**, Scatter plot for comparison of MR methods. Abbreviations: Q-test, Cochran's Q statistic heterogeneity test. MR-PRESSO, MR pleiotropy residual sum and outlier test.

For per one-unit increase in the log OR of genetic liability to colorectal adenomas, the abundance of *Gammaproteobacteria*, *Enterobacteriaceae, Fusobacteriaceae, Allisonella, RuminococcaceaeUCG013* and *Oxalobacteraceae, RuminococcaceaeUCG009* would have 0.027 (95% CI, 0.017–0.037; *P* = 7.06×10^−8^), 0.023 (95% CI, 0.013–0.034; *P* = 1.29×10^−5^), 0.006 (95% CI, 0.002–0.011; *P* = 0.006), 0.040(95% CI, 0.007–0.073; *P* = 0.021), 0.014(95% CI, 0.002–0.026; *P* = 0.018) higher log-transformed relative abundance and 0.018(95% CI, −0.036–0.001; *P* = 0.043), 0.025(95% CI, −0.039–0.011; *P* = 0.001) lower log-transformed relative abundance respectively, based on the IVW-RE model. The results for *Gammaproteobacteria*, *Enterobacteriaceae* survived the multiple test correction and were consistent across all the sensitivity analyses (Figs. 2 and 3). The MR-Egger results for *Fusobacteriaceae* and *RuminococcaceaeUCG009* were inconsistent (*β* = −0.001 and *β* = 0.012, respectively), which might indicate the existence of potential heterogeneity and directional horizontal pleiotropy, however, the *P* value for the MR-Egger intercept were 0.917 and 0.660 for these tests. The results also indicated consistency in the *β* coefficients of the associations between colorectal cancer, adenomas, and polyps with these specific bacterial taxa, implying a uniform direction of effect between benign and malignant lesions.

We observed little associations of genetic liability to colorectal polyps with other gut microbiota in the reverse MR (Supplementary Table S9). We also derived genetic IVs for colorectal cancer and adenomas combined (colorectal neoplasia), and found that per one-unit increase in the log OR of genetic liability to colorectal neoplasia was suggestively associated with 0.017 higher log-transformed relative abundance for *Gammaproteobacteria* (95% CI, 0.002–0.032; *P* = 0.023), and the result was consistent with other approaches but did not survive FDR correction (Supplementary Table S9).

## Discussion

In this research, we examined the bidirectional relationship between gut microbiome and colorectal cancer within the framework of two-sample MR. Forward MR analyses provided little support for the relationship between levels of gut microbiota taxa or metabolites and colorectal cancer risk. These findings are at odds with evidence from various sources suggesting that bacterial taxa within the microbiome cause colorectal cancer (27–29). Whilst it is not feasible to completely exclude the potential existence of a causal relationship for all taxa, we find little evidence using genetic IVs for those bacterial taxa commonly associated in previous papers. In contrast to the forward analysis, our study revealed that genetic liability to colorectal adenomas was associated with the increased abundance of the class *Gammaproteobacteria* and the family *Enterobacteriaceae* in the reverse MR conducted on MiBioGen consortium datasets. Using the summary-level data from the FR02 cohort, suggestive evidence suggested that genetic liability to colorectal adenomas was associated with the abundance of the family *Fusobacteriaceae*. According to Brenner and colleagues, the adenoma–carcinoma sequence refers that majority of colorectal cancer cases are typically preceded by dysplastic adenomas, which may advance to malignant forms (30). Piciocchi and colleagues (31) showed that cycle-inhibiting factor toxin was linked to polyps or adenomas, whereas the presence of pks+ appeared to be a predisposing factor for colorectal cancer. Bacterial toxins are capable of promoting tumorigenesis through several mechanisms, including causing DNA damage and inducing genomic instability in host cells, resistance to cell death, stimulation of signaling pathways involved in cell proliferation, and inflammation (32, 33), thus creating a favorable host cell environment for the development of colorectal cancer. Nevertheless, the association and direction of effect for benign and malignant lesions are consistent, albeit that the do not survive multiple testing for colorectal cancer. This might be due to qualitatively different proportions of the variance explained in adenomas by the genetic IVs used in these analyses compared with cancer.

*Gammaproteobacteria* is an important class of the phylum *Proteobacteria*, and *Enterobacteriaceae* is one of the major families of *Gammaproteobacteria*. Although a previous study conducted in Japanese presented that *Proteobacteria* would increase the risk of colorectal cancer (34), our forward MR analyses did not find any causal relationship of *Gammaproteobacteria* and *Enterobacteriaceae* on colorectal cancer risk in this European population, which might be attributed to the heterogeneity of different ethnic lines. Nevertheless, the reverse MR analyses revealed that genetic liability to colorectal adenomas was linked to the increased abundance of *Gammaproteobacteria* and *Enterobacteriaceae*, suggesting that the abundance of these two species are more likely to be the consequences of altered pathological environment during the etiology of a colorectal adenoma. These findings are accord with previous observational studies. Analysis of mucosal samples revealed that patients with adenoma displayed an enrichment of eight bacterial within the class *Gammaproteobacteria* (35). Moreover, compared with adjacent normal colonic mucosa, *Gammaproteobacteria* was more predominant in the adenoma mucosa (36). On the basis of stool samples, a case–control study of 144 adenoma cases, 73 serrated polyps cases, and 323 polyp-free controls also found that adenoma cases had an increased abundance of *Gammaproteobacteria* (37). For the *Enterobacteriaceae* family, the most representative genera *Escherichia* and *Shigella* had been reported more enriched in the colonic mucosa of patients with adenoma than healthy controls (38, 39). Studies revealed that strong antimicrobial bile acid activities in early-stage tumor microenvironment led to marked alterations in the composition of gut microbiota, which included a proportional upsurge in certain species of *Gammaproteobacteria* and *Bacteroidetes* species (40). *Gammaproteobacteria* was also observed to form co-exclusive network with fungal classes like *Ascomycota* phylum and participate in new interkingdom interactions in colorectal cancer (41). Because the precise mechanism underlying the augmented prevalence of *Enterobacteriaceae* among patients with adenomas remains indeterminate, it necessitates further researches to explore the interplay between *Gammaproteobacteria* and other microbiota in the development of colorectal neoplasm.

There are several strengths in our study. Firstly, we conducted a bidirectional MR study which comprehensively estimates the causal relationship between gut microbiota and colorectal cancer in both directions. This design technique minimizes confounding by known and unknown risk factors and avoids reverse causality. Moreover, we included three different stages of colorectal carcinogenesis as outcomes, which could partly reflect the effects of studied gut microbiota as well as their metabolites on the sequencing progression of colorectal neoplasia. In addition, we performed several sensitivity analyses and consistency in our results between adenomas with both *Gammaproteobacteria* and *Enterobacteriaceae* indicated that our results were robust to violations of MR assumptions.

Despite the strengths, several limitations of this study should be considered. Firstly, GWASs for gut microbiota are still in their infancy and we cannot identify enough SNPs robustly associated with the nine gut microbiota taxa of interest at the widely used genome-wide significance level (*P* < 5×10^−8^), thus we used a lenient threshold (*P* < 1×10^−5^) for the selection of genetic IVs as a less stringent analysis. There exists a strong correlation between the significance of heritability and the number of independent loci under a relaxed threshold of 1×10^−5^, which also has the greatest explanatory power with regard to microbial features (13, 16). However, using a lenient *P* value threshold of 1×10^−5^ to select IVs may result in weak instrument bias and horizontal pleiotropy. Also, given many bacteria were only represented by one or two SNPs using 5×10^−8^ threshold, the genetic explanation (R^2^) was low and may yielded inadequate statistical power for the detection of modest or minor correlations. Therefore, the causal relationships between microbiota and colorectal cancer could not be completely ruled out by the negative results of this study. Secondly, it had been acknowledged that the genetic factors involved in proximal and distal colorectal cancer are different (42), and likewise, the composition of the microbiota involved in left and right colon cancer are also distinct (43–45). We cannot exclude the potential that the null findings for colorectal cancer might be due to the mixture of left and right cases on colorectal cancer summary statistics. Thirdly, the composition of gut microbiota is subject to multifactorial influences, including lifestyle factors such as dietary patterns, medication usage, and health status. It results in the diminishing the variance explained by genetic instruments, particularly in individuals who consume a westernized diet characterized by high levels of saturated fat and red meat, and low fiber (46). We cannot exclude the possible diet–gene, gene–environment interactions, as well as nongenetic effects on outcomes, which may influence the observed results, either.

Meanwhile, confining our analysis to the individuals of European descent reduces the extent to which our findings can be extrapolated to other populations. In addition, we extracted SNPs for polyps from a candidate study (21) instead of a GWAS, so the statistical power for the analysis is constrained, underscoring the necessity for larger GWASs of colorectal neoplasia to ensure sufficient statistical power. Likewise, due to the absence of available GWAS summary data for adenoma and polyps, we have solely employed colorectal cancer as the outcome in the forward MR analysis. It should be noted that when deriving a combined set of genetic IVs from adenoma and colorectal cancer to explore the combined effect of colorectal neoplasia, the directions of associations were consistent with the effect of solely colorectal cancer but their effect estimates had wider 95% CIs. One possible reason of the wide CIs is the different power in the contributing studies. Another possible reason may be the phenotypic heterogeneities between adenomas and colorectal cancer in their relations to these bacteria. Ideally, we would incorporate large-scale microbiome and genetic data from subjects who harbour colorectal adenomas or cancer at the time of fecal sampling. However, such data are not currently available and so this is a potential shortcoming of the current analysis. Furthermore, analysis of genetic sequencing has identified genetic variations (e.g., in *LINC01605*, *PROKR2*, and *CCSER1* genes) linked to constituents of the metabolome or microbiome, as well as the risk of adenoma or colorectal cancer. In addition, the examination also discovered associations between genes responsible for cholesterol metabolism and the levels of high-density lipoprotein cholesterol would affect adenoma and colorectal cancer risk (47). Integration of metabolomics (e.g., fecal levels of cholesteryl esters and sphingolipids in colorectal cancer) and microbiome data (e.g., *Fusobacterium, Parvimonas*, and *Staphylococcus*) also indicated close interplay between bacteria and host (48). Finally, our study design is based on the assumption that the associations between gut microbiome with their metabolites and colorectal cancer risk are linear, which might veil the nonlinear effects.

In conclusion, there is not enough evidence of causal relationship between levels of gut microbiota taxa, or bacterial metabolites, and the risk of colorectal cancer based on current data. In contrast, the reverse MR provides evidence for positive associations between risk of colorectal adenomas with the abundance of the class *Gammaproteobacteria*, and the families *Enterobacteriaceae*. These findings suggest that changes in the abundance of *Gammaproteobacteria* and *Enterobacteriaceae* are potential microbial signatures during the adenoma–carcinoma sequence of colorectal cancer, while that attempts to modify the gut microbiota using measures such as antibiotic therapy and probiotics are unlikely to be successful in reducing colorectal cancer risk. This study yields insights regarding the causal relationship between colorectal carcinogenesis and gut microbiota and thus offering some reference and directions for the future study of gut microbiota.

## Supplementary Material

Supplementary Methods Supplementary Methods shows the formulas for calculating F-statistics.

Table S1 Table S1 shows the SNPs that associated with 24 microbiota taxa in MiBioGen (P<5×10-8).

Table S2 Table S2 shows the SNPs that associated with nine microbiota taxa (P<1×10-5).

Table S3 Table S3 shows the SNPs that associated with six gut microbial metabolites.

Table S4 Table S4 shows the SNPs for colorectal cancer.

Table S5 Table S5 shows the SNPs for adenoma and polyps.

Table S6 Table S6 shows the forward MR analyses of 24 gut microbiota with the risk of colorectal cancer.

Table S7 Table S7 shows the forward MR analyses of nine gut microbiota with the risk of colorectal cancer.

Table S8 Table S8 shows the forward MR analyses of the six gut microbial metabolites with the risk of colorectal cancer.

Table S9 Table S9 shows the reverse MR analyses of colorectal cancer, adenoma and polyps with the abundance of nine gut microbiota.
