# Supplementary material for: Exploring the Complex Relationship between Gut Microbiota and Risk of Colorectal Neoplasia Using Bidirectional Mendelian Randomization Analysis
Source: Cancer Epidemiol Biomarkers Prev. 2023 Apr 3;32(6):809–17. doi: 10.1158/1055-9965.EPI-22-0724 (PMC10233354; doi:10.1158/1055-9965.EPI-22-0724)
Supplement: Supplementary Methods — shows the formulas for calculating F-statistics. [file epi-22-0724_supplementary_methods_suppsm.docx]

**Supplementary Methods**

**Formulas for calculating F-statistics**

Formula (1): calculate the variance explained (*R^2^*) for determining the F-statistics.

$$R^{2}=\frac{2(MAF)(1-MAF)\beta^{2}}{2\left( MAF \right)\left( 1-MAF \right)\beta^{2}+2(N)(MAF)(1-MAF){(SE(\beta))}^{2}}$$

MAF = minor allele frequency, β = effect size, N = sample size.

Formula (2): calculate the F-statistics.

$$F=\frac{R^{2} (N-1-k)}{(1-R^{2})k}$$

R^2^ = variance explained by the SNPs calculated in (1) above, k = the number of SNP, N = sample size
