# Supplementary material for: Exploring the Complex Relationship between Gut Microbiota and Risk of Colorectal Neoplasia Using Bidirectional Mendelian Randomization Analysis
Source: Cancer Epidemiol Biomarkers Prev. 2023 Apr 3;32(6):809–17. doi: 10.1158/1055-9965.EPI-22-0724 (PMC10233354; doi:10.1158/1055-9965.EPI-22-0724)
Supplement: Table S1 — shows the SNPs that associated with 24 microbiota taxa in MiBioGen (P<5×10^-8^). [file epi-22-0724_table_s1_suppst1.docx]

**Table S1.** **The SNPs that associated with 24 microbiota taxa in MiBioGen (*P*<5×10^-8^).**

| Trait | SNP | Chr | Position | OA | EA | EAF | Beta | SE | P | N | F-statistics | R^2^ |
| --- | --- | --- | --- | --- | --- | --- | --- | --- | --- | --- | --- | --- |
| *Actinobacteria^1^* | rs998451 | 2 | 135429288 | A | G | 0.3429 | 0.06104 | 0.01159 | 3.64E-08 | 16312 | 27.74 | 0.17% |
| *Bifidobacteriaceae* | rs7322849 | 13 | 112859829 | C | T | 0.9036 | 0.11068 | 0.02010 | 1.74E-08 | 14915 | 30.32 | 0.41% |
|  | rs998451 | 2 | 135429288 | A | G | 0.3429 | 0.06801 | 0.01215 | 7.44E-09 | 14915 | 31.36 |  |
| *Oxalobacteraceae* | rs4428215 | 3 | 171947435 | A | G | 0.7396 | 0.12561 | 0.02301 | 4.88E-08 | 5167 | 29.80 | 0.57% |
| *Peptostreptococcaceae* | rs61841503 | 10 | 17019559 | A | G | 0.8628 | 0.09197 | 0.01613 | 9.80E-09 | 15333 | 32.49 | 0.21% |
| *Streptococcaceae* | rs74348118 | 12 | 100477080 | T | C | 0.0686 | -0.12405 | 0.02224 | 4.96E-08 | 15426 | 31.10 | 0.20% |
| *Eubacterium coprostanoligenes group* | rs17159861 | 7 | 31085162 | T | C | 0.1332 | 0.09622 | 0.01684 | 1.04E-08 | 17380 | 32.65 | 0.19% |
| *Ruminococcus torques group* | rs281379 | 19 | 49214274 | G | A | 0.4602 | -0.06002 | 0.01085 | 3.35E-08 | 17240 | 30.59 | 0.18% |
| *Allisonella* | rs2480213 | 9 | 79107623 | G | A | 0.2306 | 0.16708 | 0.02973 | 4.77E-08 | 3212 | 31.57 | 0.97% |
| *Bifidobacterium* | rs7322849 | 13 | 112859829 | C | T | 0.9036 | 0.11243 | 0.02018 | 1.08E-08 | 14778 | 31.03 | 0.45% |
|  | rs998451 | 2 | 135429288 | A | G | 0.3429 | 0.07274 | 0.01220 | 6.17E-10 | 14778 | 35.55 |  |
| *Enterorhabdu* | rs1355109 | 4 | 127325506 | T | C | 0.5348 | -0.09457 | 0.01629 | 7.09E-09 | 7524 | 33.67 | 0.45% |
| *Erysipelatoclostridium* | rs9914071 | 17 | 10171484 | T | C | 0.4056 | -0.08222 | 0.01441 | 1.28E-08 | 9783 | 32.54 | 0.33% |
| *Faecalibacterium* | rs12320842 | 12 | 83502773 | G | C | 0.1322 | 0.09483 | 0.01640 | 7.57E-09 | 17638 | 33.45 | 0.19% |
| *Intestinibacter* | rs10805326 | 4 | 14324623 | A | G | 0.7217 | 0.07752 | 0.01397 | 3.55E-08 | 12303 | 30.80 | 0.25% |
| *Oxalobacter* | rs736744 | 9 | 87514407 | T | C | 0.5845 | 0.11788 | 0.02113 | 2.57E-08 | 4655 | 31.12 | 0.66% |
| *Peptococcus* | rs57905414 | 3 | 17702616 | A | T | 0.8936 | 0.18482 | 0.03229 | 1.30E-08 | 5483 | 32.76 | 0.59% |
| *Romboutsia* | rs61841503 | 10 | 17019559 | A | G | 0.8628 | 0.09289 | 0.01715 | 4.00E-08 | 13568 | 29.35 | 0.22% |
| *RuminococcaceaeUCG013* | rs12781711 | 10 | 2219930 | T | C | 0.3131 | -0.06561 | 0.01175 | 2.55E-08 | 16772 | 31.19 | 0.19% |
| *RuminococcaceaeUCG009* | rs8009993 | 14 | 39306809 | C | G | 0.8708 | -0.13594 | 0.02449 | 4.42E-08 | 7389 | 30.81 | 0.42% |
| *Ruminococcus1* | rs4148901 | 11 | 45672926 | T | G | 0.4672 | -0.06058 | 0.01100 | 3.48E-08 | 16607 | 30.30 | 0.18% |
| *Streptococcus* | rs11110118 | 12 | 100302337 | C | T | 0.9225 | -0.11376 | 0.02030 | 3.28E-08 | 15634 | 31.39 | 0.20% |
| *Tyzzerella3* | rs67476743 | 19 | 1030320 | G | T | 0.7147 | 0.13216 | 0.02221 | 3.74E-09 | 4948 | 35.40 | 0.71% |
| *Bifidobacteriales* | rs7322849 | 13 | 112859829 | C | T | 0.9036 | 0.11068 | 0.02010 | 1.74E-08 | 14915 | 30.32 | 0.41% |
|  | rs998451 | 2 | 135429288 | A | G | 0.3429 | 0.06801 | 0.01215 | 7.44E-09 | 14915 | 31.36 |  |
| *Gastranaerophilales* | rs9864379 | 3 | 14306949 | C | T | 0.8867 | -0.16052 | 0.02928 | 4.66E-08 | 5768 | 30.05 | 0.52% |
| *Actinobacteria^2^* | rs6723108 | 2 | 135479980 | G | T | 0.3479 | 0.06049 | 0.01103 | 1.02E-08 | 17990 | 30.07 | 0.17% |

SNP, single nucleotide polymorphism; Chr, chromosome; OA, other allele; EA, effect allele; EAF, effect allele frequency; Beta, the estimate of the genetic association between the instrument and the exposure (i.e., log-transformed counts of the microbiota taxa); SE, standard error; N, sample size.

^1^ class *Actinobacteria*

**^2^** phylum *Actinobacteria*
