# Supplementary material for: Exploring the Complex Relationship between Gut Microbiota and Risk of Colorectal Neoplasia Using Bidirectional Mendelian Randomization Analysis
Source: Cancer Epidemiol Biomarkers Prev. 2023 Apr 3;32(6):809–17. doi: 10.1158/1055-9965.EPI-22-0724 (PMC10233354; doi:10.1158/1055-9965.EPI-22-0724)
Supplement: Table S2 — shows the SNPs that associated with nine microbiota taxa (P<1×10^-5^). [file epi-22-0724_table_s2_suppst2.docx]

| **Table S2. The SNPs that associated with** **nine microbiota taxa (*P*<1×10^-5^).** | | | | | | | | | | | | |
| --- | --- | --- | --- | --- | --- | --- | --- | --- | --- | --- | --- | --- |
| Trait | | SNP | Chr | Position | OA | EA | EAF | Beta | SE | P | N | F-statistics |
| *Gammaproteobacteria ^a^* | | rs12089016 | 1 | 186501914 | C | A | 0.1173 | -0.07875 | 0.017241 | 9.53E-06 | 15232 | 20.86 |
|  |  | rs16852925 | 2 | 167918547 | C | G | 0.83 | 0.06281 | 0.014378 | 8.85E-06 | 16019 | 19.08 |
|  |  | rs75101789 | 3 | 18615903 | T | C | 0.1561 | 0.07290 | 0.016300 | 8.79E-06 | 16024 | 20.00 |
|  |  | rs4896254 | 6 | 137612394 | A | T | 0.6203 | -0.05384 | 0.012035 | 6.43E-06 | 15650 | 20.01 |
|  |  | rs13266408 | 8 | 115095516 | T | C | 0.3917 | 0.05298 | 0.011624 | 5.78E-06 | 16025 | 20.77 |
|  |  | rs2234691 | 10 | 48385682 | C | T | 0.1541 | 0.07629 | 0.017196 | 8.39E-06 | 16011 | 19.68 |
|  |  | rs11181912 | 12 | 43572952 | A | G | 0.6551 | -0.05792 | 0.011880 | 9.95E-07 | 16017 | 23.76 |
|  |  | rs79795896 | 18 | 48185248 | G | A | 0.0537 | -0.15921 | 0.035137 | 7.92E-06 | 7577 | 20.53 |
|  |  | rs9973122 | 18 | 75633634 | A | T | 0.8579 | 0.07406 | 0.016260 | 6.77E-06 | 15650 | 20.74 |
| *Lactobacillales ^b^* | | rs57872228 | 1 | 200418805 | T | C | 0.0626 | -0.06884 | 0.014699 | 2.58E-06 | 17293 | 21.93 |
|  |  | rs1595463 | 2 | 231723657 | A | C | 0.4871 | 0.04797 | 0.010880 | 7.44E-06 | 17276 | 19.44 |
|  |  | rs1962325 | 3 | 4800550 | G | C | 0.1928 | 0.05469 | 0.011507 | 1.96E-06 | 17274 | 22.58 |
|  |  | rs11720390 | 3 | 94103591 | A | G | 0.9254 | 0.10008 | 0.022303 | 9.73E-06 | 15940 | 20.13 |
|  |  | rs74663707 | 3 | 184371224 | T | C | 0.0726 | 0.09826 | 0.022461 | 8.40E-06 | 15695 | 19.13 |
|  |  | rs80205102 | 4 | 98028407 | T | C | 0.2396 | -0.05902 | 0.012912 | 9.06E-06 | 17295 | 20.89 |
|  |  | rs111552159 | 4 | 184681021 | G | C | 0.0636 | 0.12885 | 0.028740 | 8.31E-06 | 11312 | 20.10 |
|  |  | rs113280052 | 5 | 174170255 | T | C | 0.0636 | -0.10217 | 0.022455 | 5.78E-06 | 15758 | 20.70 |
|  |  | rs9345899 | 6 | 67559323 | G | A | 0.1312 | -0.08252 | 0.017576 | 3.78E-06 | 16523 | 22.04 |
|  |  | rs78938557 | 7 | 36349586 | C | T | 0.3608 | 0.10553 | 0.023358 | 2.31E-06 | 15152 | 20.41 |
|  |  | rs2952183 | 8 | 10126062 | C | T | 0.16 | -0.06858 | 0.015099 | 3.36E-06 | 17294 | 20.63 |
|  |  | rs12797734 | 11 | 8332350 | C | T | 0.7475 | 0.05713 | 0.012711 | 7.77E-06 | 16820 | 20.20 |
|  |  | rs7959495 | 12 | 100567981 | A | C | 0.9314 | -0.10145 | 0.021948 | 6.13E-06 | 15956 | 21.36 |
|  |  | rs9581006 | 13 | 24973509 | T | C | 0.0924 | 0.22583 | 0.046923 | 1.77E-06 | 4224 | 23.15 |
|  |  | rs11627423 | 14 | 33200623 | A | C | 0.6103 | 0.04993 | 0.010974 | 5.09E-06 | 17275 | 20.69 |
|  |  | rs13379080 | 14 | 97525875 | T | C | 0.0885 | -0.08026 | 0.018083 | 8.94E-06 | 17184 | 19.70 |
|  |  | rs12448994 | 16 | 989381 | C | T | 0.841 | 0.06485 | 0.014654 | 7.63E-06 | 16525 | 19.58 |
|  |  | rs113343986 | 17 | 40778189 | G | A | 0.4274 | 0.04973 | 0.011028 | 7.52E-06 | 17293 | 20.33 |
|  |  | rs34989881 | 19 | 51959855 | G | A | 0.0656 | 0.11348 | 0.024645 | 4.09E-06 | 12921 | 21.20 |
| *Enterobacteriaceae ^c^* | | rs80319214 | 2 | 4015933 | G | C | 0.1153 | 0.09904 | 0.021588 | 6.95E-06 | 12641 | 21.04 |
|  |  | rs2374342 | 2 | 42133542 | A | C | 0.6431 | 0.05829 | 0.012619 | 4.52E-06 | 13577 | 21.33 |
|  |  | rs111229068 | 11 | 2563450 | T | A | 0.1054 | 0.11056 | 0.024172 | 3.65E-06 | 12350 | 20.92 |
|  |  | rs11026530 | 11 | 22379097 | C | T | 0.8658 | 0.08224 | 0.018638 | 9.43E-06 | 13485 | 19.47 |
|  |  | rs79757635 | 13 | 110840418 | A | C | 0.839 | 0.07586 | 0.017129 | 9.32E-06 | 13419 | 19.61 |
|  |  | rs61973590 | 14 | 27718039 | G | C | 0.2942 | -0.06054 | 0.013370 | 8.54E-06 | 13570 | 20.50 |
|  |  | rs35673018 | 16 | 54327745 | A | G | 0.9056 | 0.08996 | 0.020293 | 7.63E-06 | 13326 | 19.65 |
|  |  | rs4792380 | 17 | 13521040 | T | A | 0.0517 | 0.11560 | 0.025768 | 9.49E-06 | 12763 | 20.12 |
|  |  | rs504442 | 18 | 55145547 | G | T | 0.1392 | 0.08416 | 0.018948 | 5.17E-06 | 13578 | 19.72 |
|  |  | rs78143293 | 18 | 57672335 | G | A | 0.1441 | -0.08485 | 0.017042 | 1.20E-06 | 13572 | 24.79 |
|  |  | rs62210022 | 20 | 55336440 | C | T | 0.7843 | 0.07015 | 0.015505 | 5.30E-06 | 13513 | 20.47 |
| *Porphyromonadaceae ^d^* | | rs35961441 | 1 | 240929774 | C | A | 0.0736 | 0.09153 | 0.020731 | 8.37E-06 | 16374 | 19.49 |
|  |  | rs1125465 | 3 | 62034833 | C | T | 0.2247 | -0.05901 | 0.012249 | 1.93E-06 | 18028 | 23.21 |
|  |  | rs2675411 | 3 | 189852013 | G | A | 0.7555 | 0.05700 | 0.012128 | 3.45E-06 | 18028 | 22.09 |
|  |  | rs12486680 | 3 | 191568066 | C | A | 0.0666 | 0.09846 | 0.021857 | 4.29E-06 | 17108 | 20.29 |
|  |  | rs864093 | 4 | 149825977 | C | A | 0.2565 | -0.05279 | 0.011750 | 9.60E-06 | 18027 | 20.19 |
|  |  | rs1029811 | 7 | 69037529 | G | T | 0.1551 | 0.06733 | 0.014546 | 2.91E-06 | 18026 | 21.42 |
|  |  | rs7038649 | 9 | 2203640 | T | C | 0.2048 | -0.05806 | 0.013011 | 7.69E-06 | 18023 | 19.91 |
|  |  | rs10858364 | 9 | 138076081 | T | G | 0.2763 | 0.05532 | 0.012085 | 4.31E-06 | 18028 | 20.95 |
|  |  | rs10762312 | 10 | 71571863 | A | G | 0.7386 | -0.05237 | 0.011881 | 8.70E-06 | 18028 | 19.42 |
|  |  | rs7330827 | 13 | 23531802 | C | T | 0.9245 | -0.10384 | 0.023736 | 8.05E-06 | 13754 | 19.14 |
|  |  | rs1980561 | 14 | 63386516 | G | A | 0.6143 | -0.04853 | 0.010928 | 8.95E-06 | 18028 | 19.72 |
|  |  | rs35233670 | 17 | 63750903 | C | T | 0.5586 | -0.04736 | 0.010602 | 7.91E-06 | 18024 | 19.95 |
| *Fusobacteriaceae ^e^* | | rs34980001 | 1 | 51313536 | A | G | 0.0492 | 0.05295 | 0.011595 | 4.90E-06 | 5959 | 20.85 |
|  |  | rs61816054 | 1 | 170987700 | G | A | 0.0145 | 0.10081 | 0.020964 | 1.50E-06 | 5959 | 23.12 |
|  |  | rs330746 | 1 | 187263991 | T | C | 0.2637 | -0.02558 | 0.005686 | 6.80E-06 | 5959 | 20.23 |
|  |  | rs72753536 | 1 | 213855841 | G | A | 0.0115 | 0.11044 | 0.023575 | 2.80E-06 | 5959 | 21.94 |
|  |  | rs12029094 | 1 | 215716068 | A | G | 0.3833 | 0.02378 | 0.005189 | 4.60E-06 | 5959 | 21.00 |
|  |  | rs59437174 | 1 | 240451399 | C | G | 0.0950 | -0.03904 | 0.008782 | 8.80E-06 | 5959 | 19.76 |
|  |  | rs72802577 | 2 | 57656237 | T | C | 0.0162 | 0.08897 | 0.019874 | 7.60E-06 | 5959 | 20.03 |
|  |  | rs115079672 | 2 | 217871659 | T | C | 0.0171 | -0.08695 | 0.019504 | 8.30E-06 | 5959 | 19.87 |
|  |  | rs79720220 | 3 | 84991710 | A | G | 0.0283 | 0.06887 | 0.015207 | 5.90E-06 | 5959 | 20.50 |
|  |  | rs6438863 | 3 | 124973202 | T | G | 0.3184 | -0.02408 | 0.005364 | 7.10E-06 | 5959 | 20.15 |
|  |  | rs1872170 | 3 | 176955378 | G | T | 0.4047 | -0.02528 | 0.005178 | 1.10E-06 | 5959 | 23.82 |
|  |  | rs115148262 | 3 | 184338487 | T | G | 0.0101 | 0.11552 | 0.025084 | 4.10E-06 | 5959 | 21.20 |
|  |  | rs76691903 | 5 | 111855345 | G | A | 0.0179 | 0.08456 | 0.018983 | 8.40E-06 | 5959 | 19.84 |
|  |  | rs2431510 | 5 | 112766659 | T | C | 0.0265 | 0.07684 | 0.015755 | 1.10E-06 | 5959 | 23.78 |
|  |  | rs4469275 | 6 | 66759764 | G | A | 0.0106 | 0.11099 | 0.024602 | 6.40E-06 | 5959 | 20.35 |
|  |  | rs77137860 | 6 | 90343633 | G | A | 0.1604 | 0.03155 | 0.006960 | 5.80E-06 | 5959 | 20.54 |
|  |  | rs1476635 | 7 | 2884635 | G | A | 0.1472 | 0.03554 | 0.007182 | 7.50E-07 | 5959 | 24.47 |
|  |  | rs117995346 | 7 | 30839338 | C | T | 0.0104 | 0.11398 | 0.025043 | 5.30E-06 | 5959 | 20.71 |
|  |  | rs62473940 | 7 | 115148174 | T | C | 0.0222 | 0.07663 | 0.017199 | 8.40E-06 | 5959 | 19.84 |
|  |  | rs11781594 | 8 | 18974925 | A | C | 0.1423 | 0.03227 | 0.007282 | 9.40E-06 | 5959 | 19.62 |
|  |  | rs11224464 | 11 | 100812285 | T | C | 0.0852 | 0.04438 | 0.009006 | 8.30E-07 | 5959 | 24.28 |
|  |  | rs4367998 | 12 | 2576758 | T | C | 0.9622 | 0.07073 | 0.015593 | 5.70E-06 | 5959 | 20.57 |
|  |  | rs77318370 | 12 | 72138721 | G | A | 0.0433 | -0.05602 | 0.012377 | 6.00E-06 | 5959 | 20.48 |
|  |  | rs180898386 | 16 | 9019442 | T | C | 0.0119 | 0.10497 | 0.023097 | 5.50E-06 | 5959 | 20.65 |
|  |  | rs111838219 | 17 | 43581904 | A | G | 0.0113 | 0.11173 | 0.024045 | 3.40E-06 | 5959 | 21.58 |
|  |  | rs112545520 | 20 | 4738175 | C | T | 0.0126 | 0.10181 | 0.022787 | 7.90E-06 | 5959 | 19.96 |
|  |  | rs6003629 | 22 | 23331106 | C | A | 0.3389 | -0.02486 | 0.005499 | 6.20E-06 | 5959 | 20.42 |
|  |  | rs117340124 | 22 | 34967440 | A | G | 0.0148 | 0.09550 | 0.020857 | 4.70E-06 | 5959 | 20.96 |
| *Actinomyces ^f^* | | rs71315246 | 3 | 101352439 | G | A | 0.1431 | -0.09698 | 0.021925 | 9.83E-06 | 7468 | 19.56 |
|  |  | rs34583783 | 6 | 67207371 | T | G | 0.1024 | 0.12660 | 0.026846 | 4.49E-06 | 7468 | 22.23 |
|  |  | rs35011108 | 6 | 133007480 | G | A | 0.0636 | 0.23263 | 0.051204 | 6.34E-06 | 3515 | 20.63 |
|  |  | rs4073240 | 6 | 169224781 | A | G | 0.3827 | 0.07497 | 0.016737 | 7.94E-06 | 7468 | 20.06 |
|  |  | rs4146653 | 10 | 4782841 | A | G | 0.1968 | 0.09852 | 0.021418 | 4.50E-06 | 7468 | 21.15 |
|  |  | rs10787984 | 10 | 121356829 | C | G | 0.8241 | 0.09432 | 0.021351 | 9.62E-06 | 7468 | 19.51 |
|  |  | rs4962491 | 10 | 127520723 | T | C | 0.0616 | 0.16932 | 0.036689 | 6.70E-06 | 6347 | 21.29 |
|  |  | rs2715439 | 15 | 99492313 | T | C | 0.5457 | 0.07467 | 0.016482 | 6.27E-06 | 7468 | 20.52 |
| *Bifidobacterium ^g^* | | rs12022129 | 1 | 207003374 | A | G | 0.7793 | 0.06194 | 0.013894 | 8.00E-06 | 14776 | 19.87 |
|  |  | rs4567981 | 2 | 49906864 | A | T | 0.5606 | 0.05621 | 0.011792 | 1.93E-06 | 14666 | 22.72 |
|  |  | rs479980 | 2 | 135284038 | A | C | 0.6551 | -0.06042 | 0.012291 | 8.05E-07 | 14019 | 24.16 |
|  |  | rs1961273 | 2 | 137602926 | T | C | 0.2714 | 0.06740 | 0.013232 | 3.51E-07 | 14778 | 25.95 |
|  |  | rs17241561 | 2 | 189800385 | G | T | 0.7177 | -0.05897 | 0.013126 | 8.14E-06 | 14778 | 20.18 |
|  |  | rs13020688 | 2 | 192878532 | A | G | 0.6471 | 0.05627 | 0.012262 | 4.07E-06 | 14666 | 21.06 |
|  |  | rs62287778 | 4 | 1515208 | T | C | 0.3867 | 0.05338 | 0.012072 | 9.76E-06 | 14776 | 19.55 |
|  |  | rs56302516 | 5 | 518434 | C | T | 0.7366 | 0.06075 | 0.013505 | 6.40E-06 | 14666 | 20.23 |
|  |  | rs73797465 | 5 | 142793467 | G | T | 0.9135 | -0.09536 | 0.020924 | 4.38E-06 | 14666 | 20.77 |
|  |  | rs41389446 | 5 | 169747593 | G | T | 0.1243 | -0.08228 | 0.018490 | 8.22E-06 | 13806 | 19.80 |
|  |  | rs857444 | 6 | 14617591 | T | C | 0.6392 | 0.05582 | 0.012122 | 3.57E-06 | 14777 | 21.20 |
|  |  | rs2686789 | 7 | 48090640 | C | A | 0.1153 | 0.07065 | 0.015793 | 7.65E-06 | 14778 | 20.01 |
|  |  | rs2491158 | 10 | 126089703 | A | G | 0.8181 | 0.07126 | 0.015983 | 8.05E-06 | 14021 | 19.88 |
|  |  | rs10841473 | 12 | 20378911 | C | G | 0.7296 | -0.06242 | 0.012944 | 1.65E-06 | 14777 | 23.25 |
|  |  | rs7322849 | 13 | 112859829 | C | T | 0.9036 | 0.11243 | 0.020181 | 1.08E-08 | 14778 | 31.03 |
|  |  | rs540489 | 17 | 72897722 | G | T | 0.7664 | -0.06376 | 0.013875 | 5.19E-06 | 14561 | 21.12 |
|  |  | rs117761464 | 21 | 31847241 | G | A | 0.0507 | 0.23069 | 0.050320 | 4.94E-06 | 3856 | 21.01 |
|  |  | rs5746486 | 22 | 18354272 | C | T | 0.6461 | -0.05362 | 0.012080 | 9.00E-06 | 14778 | 19.70 |
| *Roseburia ^h^* | | rs12740451 | 1 | 95178974 | C | T | 0.1252 | 0.06975 | 0.015361 | 7.34E-06 | 17837 | 20.62 |
|  |  | rs147990086 | 1 | 145655256 | G | A | 0.1909 | -0.05789 | 0.013240 | 8.93E-06 | 17840 | 19.12 |
|  |  | rs59070623 | 1 | 194182280 | T | C | 0.1193 | -0.07754 | 0.017811 | 9.34E-06 | 16945 | 18.95 |
|  |  | rs6445851 | 3 | 57116228 | A | G | 0.5994 | -0.04973 | 0.010816 | 3.53E-06 | 17851 | 21.14 |
|  |  | rs116270582 | 4 | 119349 | A | T | 0.9513 | -0.15384 | 0.032932 | 1.20E-06 | 9099 | 21.82 |
|  |  | rs2935500 | 5 | 89591470 | A | T | 0.5398 | 0.04754 | 0.010677 | 9.17E-06 | 17854 | 19.83 |
|  |  | rs28040 | 5 | 96018066 | C | G | 0.2127 | 0.05657 | 0.012679 | 9.26E-06 | 17854 | 19.90 |
|  |  | rs329182 | 5 | 125076494 | C | T | 0.8429 | 0.06903 | 0.015288 | 5.90E-06 | 17854 | 20.39 |
|  |  | rs78753150 | 5 | 144135655 | C | A | 0.0586 | 0.09687 | 0.021407 | 9.98E-06 | 16303 | 20.48 |
|  |  | rs6930661 | 6 | 12774611 | T | C | 0.0755 | -0.09616 | 0.020497 | 2.48E-06 | 16945 | 22.01 |
|  |  | rs75326254 | 6 | 166170836 | T | C | 0.0636 | -0.10463 | 0.023090 | 7.50E-06 | 15893 | 20.53 |
|  |  | rs12412854 | 10 | 16039509 | C | G | 0.5905 | 0.04723 | 0.010642 | 9.50E-06 | 17847 | 19.69 |
|  |  | rs16910295 | 11 | 12009569 | C | T | 0.9394 | -0.09804 | 0.020957 | 2.91E-06 | 16648 | 21.88 |
|  |  | rs2160994 | 12 | 50650057 | C | T | 0.6203 | 0.05507 | 0.011248 | 9.70E-07 | 17444 | 23.97 |
|  |  | rs9300744 | 13 | 103117486 | T | C | 0.2386 | -0.05885 | 0.012623 | 4.75E-06 | 17854 | 21.73 |
|  |  | rs57466170 | 15 | 37859065 | T | C | 0.0865 | 0.07414 | 0.017160 | 8.30E-06 | 17333 | 18.66 |
|  |  | rs55858165 | 15 | 86512809 | C | A | 0.0775 | 0.17928 | 0.040495 | 9.99E-06 | 5447 | 19.59 |
|  |  | rs2034590 | 16 | 8307248 | C | T | 0.2366 | 0.05794 | 0.012290 | 3.51E-06 | 17843 | 22.22 |
| *Peptostreptococcaceae ^i^* | | rs12993096 | 2 | 67823472 | C | T | 0.7654 | -0.05683 | 0.012817 | 6.31E-06 | 15981 | 19.66 |
|  |  | rs1098907 | 3 | 151754930 | A | T | 0.4284 | 0.05202 | 0.011254 | 3.88E-06 | 16060 | 21.36 |
|  |  | rs17661170 | 4 | 171175320 | A | G | 0.7366 | 0.06334 | 0.012685 | 5.95E-07 | 16061 | 24.93 |
|  |  | rs10805326 | 4 | 14324623 | A | G | 0.7217 | 0.05666 | 0.012276 | 4.03E-06 | 16061 | 21.3 |
|  |  | rs76982728 | 7 | 44312824 | C | T | 0.9612 | 0.12420 | 0.026692 | 3.24E-06 | 11185 | 21.65 |
|  |  | rs117644882 | 7 | 46708427 | C | T | 0.9364 | 0.17783 | 0.037792 | 2.80E-06 | 6629 | 22.14 |
|  |  | rs2644627 | 8 | 26726641 | G | C | 0.5636 | -0.05585 | 0.011224 | 6.33E-07 | 16059 | 24.76 |
|  |  | rs12377846 | 9 | 16786784 | A | C | 0.9513 | -0.25200 | 0.051163 | 7.26E-07 | 3736 | 24.25 |
|  |  | rs2883972 | 10 | 16976618 | A | G | 0.836 | 0.07347 | 0.014982 | 9.80E-07 | 15333 | 24.05 |
|  |  | rs77540684 | 10 | 14650535 | G | T | 0.9483 | 0.10673 | 0.024564 | 8.14E-06 | 13495 | 18.87 |
|  |  | rs9573928 | 13 | 77320869 | T | C | 0.1839 | -0.06443 | 0.014247 | 7.01E-06 | 16060 | 20.45 |
|  |  | rs59865771 | 16 | 89246670 | T | C | 0.2813 | -0.05738 | 0.012719 | 7.69E-06 | 16040 | 20.35 |
|  |  | rs1467258 | 17 | 38216487 | A | G | 0.833 | 0.07258 | 0.016232 | 7.90E-06 | 15584 | 19.99 |
|  |  | rs75819860 | 18 | 65324280 | A | T | 0.9225 | 0.12626 | 0.026838 | 2.49E-06 | 11697 | 22.13 |
|  |  | rs12986312 | 19 | 17414138 | G | T | 0.6779 | 0.05718 | 0.012595 | 5.77E-06 | 15970 | 20.61 |
|  |  | rs59987323 | 22 | 37976422 | T | C | 0.4274 | -0.05371 | 0.011443 | 2.70E-06 | 15584 | 22.03 |
|  | ^a^ R^2^ of *Gammaproteobacteria* was 1.31%. | | | | | | | | | | | |
|  | ^b^ R^2^ of *Lactobacillales* was 2.87%. | | | | | | | | | | | |
|  | ^c^ R^2^ of *Enterobacteriaceae* was 1.88%. | | | | | | | | | | | |
|  | ^d^ R^2^ of *Porphyromonadaceae* was 1.41%. | | | | | | | | | | | |
|  | ^e^ R^2^ of *Fusobacteriaceae* was 9.88%. | | | | | | | | | | | |
|  | ^f^ R^2^ of *Actinomyces* was 2.56%. | | | | | | | | | | | |
|  | ^g^ R^2^ of *Bifidobacterium* was 3.08%. | | | | | | | | | | | |
|  | ^h^ R^2^ of *Roseburia* was 2.50%. | | | | | | | | | | | |
|  | ^I^ R^2^ of *Peptostreptococcaceae* was 3.01%. | | | | | | | | | | | |

SNP, single nucleotide polymorphism; Chr, chromosome; OA, other allele; EA, effect allele; EAF, effect allele frequency; Beta, the estimate of the genetic association between the instrument and the exposure (i.e., center-log-ratio transformed abundance of *Fusobacteriaceae* and log-transformed counts of the other eight microbiota taxa); SE, standard error; N, sample size.
