# Supplementary material for: Exploring the Complex Relationship between Gut Microbiota and Risk of Colorectal Neoplasia Using Bidirectional Mendelian Randomization Analysis
Source: Cancer Epidemiol Biomarkers Prev. 2023 Apr 3;32(6):809–17. doi: 10.1158/1055-9965.EPI-22-0724 (PMC10233354; doi:10.1158/1055-9965.EPI-22-0724)
Supplement: Table S3 — shows the SNPs that associated with six gut microbial metabolites. [file epi-22-0724_table_s3_suppst3.docx]

| **Table S3. The SNPs that associated with six gut microbial metabolites.** | | | | | | | | | | | | |
| --- | --- | --- | --- | --- | --- | --- | --- | --- | --- | --- | --- | --- |
| Metabolites | SNP | Chr | | Position | Closest gene | OA | EA | MAF | Beta | SE | P | F-statistics |
| Choline ^a^ | rs10098425 | 8 | | 106016051 | ZFPM2 | A | G | 0.054 | 0.46 | 0.10 | 8.93E-06 | 21.14 |
|  | rs4008155 | 9 | | 38478919 | ALDH1B1 | C | T | 0.156 | 0.22 | 0.05 | 4.02E-06 | 19.34 |
|  | rs10819950 | 9 | | 97975154 | SLC35D2 | A | G | 0.316 | 0.21 | 0.05 | 6.95E-06 | 17.62 |
|  | rs10826197 | 10 | | 59912647 | TFAM | C | A | 0.487 | 0.15 | 0.03 | 8.15E-06 | 24.98 |
|  | rs2408564 | 12 | | 90546484 | BTG1 | A | G | 0.170 | -0.25 | 0.05 | 1.41E-06 | 24.98 |
|  | rs12444044 | 16 | | 78024057 | WWOX | A | G | 0.112 | -0.36 | 0.08 | 5.77E-06 | 20.23 |
| Betaine ^b^ | rs10178297 | 2 | | 103998526 | MRPS9 | T | C | 0.16 | 0.21 | 0.05 | 6.74E-06 | 17.62 |
|  | rs1868264 | 2 | | 239151212 | TRAF3IP1 | A | G | 0.195 | 0.19 | 0.04 | 4.92E-06 | 22.54 |
|  | rs6862283 | 5 | | 78355394 | ARSB | T | C | 0.306 | -0.19 | 0.04 | 9.57E-08 | 22.54 |
|  | rs11742447 | 5 | | 117598798 | DMXL1 | C | T | 0.345 | 0.25 | 0.05 | 1.16E-06 | 24.98 |
|  | rs10817686 | 9 | | 116739671 | TNC | T | C | 0.471 | 0.46 | 0.10 | 1.68E-06 | 21.14 |
|  | rs10786317 | 10 | | 83003759 | NRG3 | A | G | 0.437 | -0.16 | 0.04 | 4.72E-06 | 15.98 |
|  | rs10883712 | 10 | | 103929295 | PPRC1 | T | G | 0.404 | 0.16 | 0.04 | 4.02E-06 | 15.98 |
|  | rs11030909 | 11 | | 29933817 | FSHB | C | A | 0.089 | -0.29 | 0.06 | 7.30E-06 | 23.34 |
|  | rs1321958 | 11 | | 31005259 | DCDC1 | G | A | 0.388 | 0.15 | 0.03 | 8.12E-06 | 24.98 |
|  | rs2087307 | 12 | | 10153542 | CLEC1A | T | G | 0.259 | -0.17 | 0.04 | 8.63E-06 | 18.05 |
|  | rs2879414 | 18 | | 47962958 | MEX3C | A | G | 0.459 | -0.15 | 0.03 | 5.03E-06 | 24.98 |
|  | rs17815398 | 18 | | 47967754 | MEX3C | A | G | 0.020 | 0.84 | 0.19 | 7.19E-06 | 19.53 |
|  | rs358538 | 19 | | 37696665 | PDCD5 | A | G | 0.055 | 0.46 | 0.10 | 7.86E-06 | 21.14 |
| Carnitine ^c^ | rs4656852 | 1 | | 158071277 | FCRL6 | T | C | 0.053 | -0.51 | 0.11 | 8.30E-06 | 21.48 |
|  | rs7606454 | 2 | | 45795867 | SRBD1 | C | T | 0.139 | 0.24 | 0.05 | 9.62E-06 | 23.02 |
|  | rs9879988 | 3 | | 5284556 | ARL8B | A | G | 0.163 | 0.24 | 0.05 | 3.00E-07 | 23.02 |
|  | rs11733138 | 4 | | 22769759 | LOC643751 | G | A | 0.346 | 0.23 | 0.05 | 6.10E-06 | 21.14 |
|  | rs274554 | 5 | | 131752849 | LOC441108 | C | T | 0.159 | -0.21 | 0.05 | 4.68E-06 | 17.62 |
|  | rs6959875 | 7 | | 19788530 | TWISTNB | G | A | 0.301 | 0.27 | 0.06 | 7.58E-07 | 20.23 |
|  | rs16913790 | 10 | | 61016188 | CCDC6 | G | A | 0.138 | 0.22 | 0.05 | 7.18E-06 | 19.34 |
|  | rs1171617 | 10 | | 61137188 | CCDC6 | T | G | 0.230 | -0.42 | 0.04 | 5.87E-26 | 110.14 |
|  | rs1171610 | 10 | | 61147703 | CCDC6 | T | C | 0.383 | -0.2 | 0.04 | 6.63E-08 | 24.98 |
|  | rs10821590 | 10 | | 61197033 | SLC16A9 | G | A | 0.071 | 0.6 | 0.11 | 8.99E-08 | 29.72 |
|  | rs562672 | 11 | | 125488674 | RPUSD4 | T | C | 0.366 | -0.23 | 0.05 | 7.39E-06 | 21.14 |
|  | rs6108228 | 20 | | 8926063 | PLCB1 | T | G | 0.406 | -0.18 | 0.04 | 2.56E-06 | 20.23 |
| TMAO ^d^ | rs3935570 | 1 | | 19039958 | ALDH4A1 | G | T | 0.261 | -0.23 | 0.04 | 1.56E-07 | 33.03 |
|  | rs4846884 | 1 | | 229153698 | ARV1 | C | A | 0.156 | -0.21 | 0.05 | 6.19E-06 | 17.62 |
|  | rs17418675 | 2 | | 55064109 | FLJ31438 | T | C | 0.341 | 0.16 | 0.04 | 9.11E-06 | 15.98 |
|  | rs13236413 | 7 | | 156563826 | MNX1 | C | T | 0.090 | 0.41 | 0.09 | 2.23E-06 | 20.73 |
|  | rs2703228 | 8 | | 5863417 | ANGPT2 | C | G | 0.323 | -0.22 | 0.04 | 1.28E-07 | 30.22 |
|  | rs16917161 | 8 | | 53128610 | PCMTD1 | T | A | 0.092 | 0.26 | 0.06 | 9.23E-06 | 18.76 |
|  | rs12221306 | 10 | | 17041344 | RSU1 | A | T | 0.387 | -0.16 | 0.04 | 5.04E-06 | 15.98 |
|  | rs7302178 | 12 | | 26899160 | C12orf11 | A | G | 0.164 | 0.24 | 0.05 | 6.40E-06 | 23.02 |
| GABA ^e^ | rs12073624 | 1 | | 79235744 | IFI44 | G | A | 0.118 | -0.28 | 0.06 | 3.56E-06 | 21.76 |
|  | rs2553640 | 1 | | 163733501 | RXRG | G | A | 0.09 | -0.31 | 0.07 | 9.38E-06 | 19.59 |
|  | rs2912200 | 4 | | 13752463 | NKX3-2 | T | C | 0.223 | 0.2 | 0.04 | 3.39E-06 | 24.98 |
|  | rs6977081 | 7 | | 150173448 | TMEM176A | G | T | 0.336 | 0.23 | 0.04 | 5.36E-09 | 33.03 |
|  | rs927428 | 10 | | 123926834 | BTBD16 | G | A | 0.042 | -0.39 | 0.09 | 8.54E-06 | 18.76 |
|  | rs201762 | 13 | | 49963837 | RNASEH2B | T | G | 0.233 | -0.19 | 0.04 | 6.88E-06 | 22.54 |
|  | rs17587049 | 14 | | 100761050 | DLK1 | C | A | 0.196 | 0.77 | 0.15 | 2.96E-07 | 26.33 |
|  | rs8051603 | 16 | | 85606059 | MAP1LC3B | C | A | 0.039 | -0.58 | 0.13 | 9.13E-06 | 19.89 |
|  | rs8106338 | 19 | | 2054186 | C19orf36 | T | C | 0.020 | 1.97 | 0.42 | 2.81E-06 | 21.98 |
|  | rs2161525 | 19 | | 7738664 | CD209 | T | C | 0.425 | 0.19 | 0.04 | 1.00E-05 | 22.54 |
| propionic acid ^f^ | rs6766484 | 3 | | 186950433 | IGF2BP2 | C | T | 0.007 | 1.41 | 0.32 | 7.52E-06 | 19.40 |
|  | rs11132322 | 4 | | 169713165 | FLJ31033 | C | T | 0.311 | -0.21 | 0.04 | 1.22E-06 | 27.54 |
| ^a^ R^2^ of choline was 6.12%. | | |  |  |  |  |  |  |  |  |  |  |
| ^b^ R^2^ of betaine was 13.02%. | | |  |  |  |  |  |  |  |  |  |  |
| ^c^ R^2^ of carnitine was 16.58%. | | |  |  |  |  |  |  |  |  |  |  |
| ^d^ R^2^ of TMAO was 8.36%. | | |  |  |  |  |  |  |  |  |  |  |
| ^e^ R^2^ of GABA was 11.03%. | | |  |  |  |  |  |  |  |  |  |  |
| ^f^ R^2^ of propionic acid was 2.24%. | | |  |  |  |  |  |  |  |  |  |  |

SNP, single nucleotide polymorphism; Chr, chromosome; OA, other allele; EA, effect allele; MAF, minor allele frequency; Beta, the estimate of the genetic association between the instrument and the exposure (i.e., log-transformed six plasma gut microbial metabolites concentrations (i.e., μM); SE, standard error; TMAO, trimethylamine N-oxide; GABA, gamma-aminobutyric acid.
