# Supplementary material for: Exploring the Complex Relationship between Gut Microbiota and Risk of Colorectal Neoplasia Using Bidirectional Mendelian Randomization Analysis
Source: Cancer Epidemiol Biomarkers Prev. 2023 Apr 3;32(6):809–17. doi: 10.1158/1055-9965.EPI-22-0724 (PMC10233354; doi:10.1158/1055-9965.EPI-22-0724)
Supplement: Table S4 — shows the SNPs for colorectal cancer. [file epi-22-0724_table_s4_suppst4.docx]

| **Table S4. The SNPs for colorectal cancer.** | | | | | | | | | | | | |
| --- | --- | --- | --- | --- | --- | --- | --- | --- | --- | --- | --- | --- |
| Trait | SNP | Chr | Position | EA | OA | EAF | Beta | SE | P | F-statistics | Reference | Source |
| CRC | rs12144319 | 1 | 55246035 | C | T | 0.25 | 0.071 | 0.018 | 7.64E-05 | 15.64 | Huyghe et al. | Law et al. |
|  | rs6678517 | 1 | 183002639 | A | G | 0.42 | -0.087 | 0.015 | 5.86E-09 | 33.88 | Huyghe et al. | Law et al. |
|  | rs17011141 | 1 | 222112634 | G | A | 0.22 | 0.095 | 0.018 | 7.80E-08 | 28.85 | Huyghe et al. | Law et al. |
|  | rs448513 | 2 | 159964552 | C | T | 0.35 | 0.063 | 0.015 | 4.96E-05 | 16.46 | Huyghe et al. | Law et al. |
|  | rs11884596 | 2 | 199612407 | C | T | 0.39 | 0.064 | 0.016 | 4.22E-05 | 16.77 | Huyghe et al. | Law et al. |
|  | rs983402 | 2 | 199781586 | T | C | 0.65 | -0.059 | 0.015 | 1.13E-04 | 14.91 | Huyghe et al. | Law et al. |
|  | rs72942485 | 3 | 112999560 | G | A | 0.02 | -0.237 | 0.056 | 2.52E-05 | 17.75 | Huyghe et al. | Law et al. |
|  | rs10049390 | 3 | 133701119 | A | G | 0.73 | 0.067 | 0.017 | 1.09E-04 | 14.98 | Huyghe et al. | Law et al. |
|  | rs35470271 | 3 | 40915239 | G | A | 0.16 | 0.100 | 0.020 | 7.78E-07 | 24.41 | Huyghe et al. | Law et al. |
|  | rs13149359 | 4 | 94938618 | A | C | 0.37 | 0.038 | 0.015 | 1.17E-02 | 6.36 | Huyghe et al. | Law et al. |
|  | rs4976270 | 5 | 134467220 | C | T | 0.43 | -0.056 | 0.015 | 1.38E-04 | 14.53 | Huyghe et al. | Law et al. |
|  | rs7708610 | 5 | 40102443 | A | G | 0.36 | 0.029 | 0.015 | 5.73E-02 | 3.61 | Huyghe et al. | Law et al. |
|  | rs12514517 | 5 | 40280076 | A | G | 0.28 | 0.088 | 0.016 | 4.71E-08 | 29.83 | Huyghe et al. | Law et al. |
|  | rs2516420 | 6 | 31449620 | C | T | 0.07 | -0.080 | 0.030 | 8.25E-03 | 6.98 | Huyghe et al. | Law et al. |
|  | rs62404966 | 6 | 55712124 | C | T | 0.24 | -0.072 | 0.017 | 2.65E-05 | 17.65 | Huyghe et al. | Law et al. |
|  | rs12672022 | 7 | 45136423 | T | C | 0.17 | -0.054 | 0.020 | 6.36E-03 | 7.45 | Huyghe et al. | Law et al. |
|  | rs3133285 | 8 | 117629411 | G | C | 0.17 | -0.071 | 0.020 | 3.35E-04 | 12.86 | Huyghe et al. | Law et al. |
|  | rs6983267 | 8 | 128413305 | G | T | 0.47 | -0.169 | 0.015 | 5.52E-31 | 133.97 | Huyghe et al. | Law et al. |
|  | rs4313119 | 8 | 128571855 | G | T | 0.26 | -0.050 | 0.017 | 3.32E-03 | 8.62 | Huyghe et al. | Law et al. |
|  | rs34405347 | 9 | 101679752 | T | G | 0.09 | -0.072 | 0.027 | 7.13E-03 | 7.24 | Huyghe et al. | Law et al. |
|  | rs10980628 | 9 | 113671403 | C | T | 0.21 | 0.038 | 0.018 | 3.44E-02 | 4.48 | Huyghe et al. | Law et al. |
|  | rs10821907 | 10 | 52648454 | C | T | 0.19 | -0.052 | 0.019 | 7.37E-03 | 7.18 | Huyghe et al. | Law et al. |
|  | rs12246635 | 10 | 114288619 | C | T | 0.10 | 0.115 | 0.025 | 4.25E-06 | 21.15 | Huyghe et al. | Law et al. |
|  | rs704017 | 10 | 80819132 | G | A | 0.61 | 0.098 | 0.017 | 5.82E-09 | 33.89 | Huyghe et al. | Law et al. |
|  | rs2186607 | 11 | 101656397 | T | A | 0.49 | -0.034 | 0.015 | 2.09E-02 | 5.33 | Huyghe et al. | Law et al. |
|  | rs174533 | 11 | 61549025 | G | A | 0.34 | -0.056 | 0.015 | 2.96E-04 | 13.09 | Huyghe et al. | Law et al. |
|  | rs7121958 | 11 | 74280012 | G | T | 0.51 | 0.096 | 0.015 | 5.44E-11 | 43.01 | Huyghe et al. | Law et al. |
|  | rs11610543 | 12 | 43134191 | G | A | 0.48 | 0.046 | 0.015 | 1.74E-03 | 9.80 | Huyghe et al. | Law et al. |
|  | rs12372718 | 12 | 51171090 | G | A | 0.39 | 0.093 | 0.015 | 5.40E-10 | 38.52 | Huyghe et al. | Law et al. |
|  | rs597808 | 12 | 111973358 | G | A | 0.53 | 0.094 | 0.015 | 1.85E-10 | 40.61 | Huyghe et al. | Law et al. |
|  | rs7300312 | 12 | 115890922 | C | T | 0.60 | 0.080 | 0.015 | 8.84E-08 | 28.61 | Huyghe et al. | Law et al. |
|  | rs35808169 | 12 | 4368607 | C | T | 0.20 | 0.082 | 0.022 | 1.73E-04 | 14.10 | Huyghe et al. | Law et al. |
|  | rs3217874 | 12 | 4400808 | T | C | 0.44 | 0.065 | 0.015 | 1.24E-05 | 19.10 | Huyghe et al. | Law et al. |
|  | rs4759277 | 12 | 57533690 | A | C | 0.36 | 0.028 | 0.015 | 6.27E-02 | 3.46 | Huyghe et al. | Law et al. |
|  | rs2250430 | 12 | 6421174 | T | A | 0.69 | 0.055 | 0.019 | 3.53E-03 | 8.51 | Huyghe et al. | Law et al. |
|  | rs7333607 | 13 | 37462010 | G | A | 0.24 | 0.094 | 0.018 | 1.15E-07 | 28.10 | Huyghe et al. | Law et al. |
|  | rs35107139 | 14 | 54419106 | C | A | 0.40 | 0.095 | 0.016 | 2.77E-09 | 35.34 | Huyghe et al. | Law et al. |
|  | rs7160450 | 14 | 54457231 | T | C | 0.62 | -0.051 | 0.015 | 6.61E-04 | 11.59 | Huyghe et al. | Law et al. |
|  | rs17094983 | 14 | 59189361 | G | A | 0.12 | -0.042 | 0.023 | 6.02E-02 | 3.53 | Huyghe et al. | Law et al. |
|  | rs12149163 | 16 | 86339315 | T | C | 0.48 | -0.046 | 0.015 | 1.81E-03 | 9.73 | Huyghe et al. | Law et al. |
|  | rs9924886 | 16 | 68743939 | A | C | 0.26 | -0.055 | 0.017 | 1.12E-03 | 10.62 | Huyghe et al. | Law et al. |
|  | rs1078643 | 17 | 10707241 | A | G | 0.78 | 0.101 | 0.019 | 1.30E-07 | 27.86 | Huyghe et al. | Law et al. |
|  | rs983318 | 17 | 70413253 | A | G | 0.26 | 0.053 | 0.017 | 1.71E-03 | 9.83 | Huyghe et al. | Law et al. |
|  | rs4968127 | 17 | 809643 | G | A | 0.62 | -0.057 | 0.015 | 1.36E-04 | 14.55 | Huyghe et al. | Law et al. |
|  | rs75954926 | 17 | 81061048 | G | A | 0.66 | 0.064 | 0.024 | 7.27E-03 | 7.20 | Huyghe et al. | Law et al. |
|  | rs11874392 | 18 | 46453156 | A | T | 0.49 | -0.022 | 0.015 | 1.36E-01 | 2.23 | Huyghe et al. | Law et al. |
|  | rs34797592 | 19 | 16417198 | T | C | 0.11 | 0.078 | 0.026 | 2.91E-03 | 8.86 | Huyghe et al. | Law et al. |
|  | rs28840750 | 19 | 33519927 | T | G | 0.05 | -0.210 | 0.035 | 2.46E-09 | 35.57 | Huyghe et al. | Law et al. |
|  | rs73068325 | 19 | 59079096 | T | C | 0.19 | 0.067 | 0.019 | 4.13E-04 | 12.47 | Huyghe et al. | Law et al. |
|  | rs11087784 | 20 | 7740976 | G | A | 0.15 | 0.100 | 0.021 | 3.07E-06 | 21.77 | Huyghe et al. | Law et al. |
|  | rs189583 | 20 | 6376457 | G | C | 0.67 | -0.115 | 0.015 | 1.16E-13 | 55.06 | Huyghe et al. | Law et al. |
|  | rs28488 | 20 | 6762221 | T | C | 0.63 | 0.037 | 0.015 | 1.60E-02 | 5.80 | Huyghe et al. | Law et al. |
|  | rs72647484 | 1 | 22587728 | T | C | 0.09 | -0.111 | 0.026 | 2.26E-05 | 17.96 | Law et al. | Law et al. |
|  | rs61776719 | 1 | 38461319 | C | A | 0.56 | -0.068 | 0.016 | 1.56E-05 | 18.66 | Law et al. | Law et al. |
|  | rs13020391 | 2 | 219184436 | C | T | 0.36 | -0.091 | 0.015 | 4.04E-09 | 34.60 | Law et al. | Law et al. |
|  | rs11692435 | 2 | 98275354 | G | A | 0.11 | -0.110 | 0.025 | 1.09E-05 | 19.34 | Law et al. | Law et al. |
|  | rs9831861 | 3 | 53088285 | G | T | 0.59 | 0.063 | 0.015 | 2.16E-05 | 18.04 | Law et al. | Law et al. |
|  | rs12635946 | 3 | 112916918 | C | T | 0.38 | -0.084 | 0.015 | 5.33E-08 | 29.59 | Law et al. | Law et al. |
|  | rs17035289 | 4 | 106048291 | T | C | 0.18 | -0.100 | 0.019 | 2.08E-07 | 26.96 | Law et al. | Law et al. |
|  | rs75686861 | 4 | 145621328 | A | G | 0.10 | 0.113 | 0.025 | 7.55E-06 | 20.05 | Law et al. | Law et al. |
|  | rs77776598 | 5 | 1240998 | C | T | 0.07 | 0.132 | 0.032 | 4.06E-05 | 16.84 | Law et al. | Law et al. |
|  | rs2070699 | 6 | 12292772 | T | G | 0.48 | 0.064 | 0.015 | 2.22E-05 | 17.99 | Law et al. | Law et al. |
|  | rs3131043 | 6 | 30758466 | G | A | 0.43 | 0.044 | 0.019 | 2.09E-02 | 5.34 | Law et al. | Law et al. |
|  | rs16878812 | 6 | 35569562 | A | G | 0.11 | -0.103 | 0.023 | 1.02E-05 | 19.48 | Law et al. | Law et al. |
|  | rs6933790 | 6 | 41672769 | T | C | 0.17 | -0.076 | 0.021 | 3.45E-04 | 12.81 | Law et al. | Law et al. |
|  | rs6928864 | 6 | 105966894 | C | A | 0.09 | -0.124 | 0.026 | 2.72E-06 | 22.00 | Law et al. | Law et al. |
|  | rs1321310 | 6 | 36623124 | C | T | 0.24 | 0.076 | 0.017 | 8.95E-06 | 19.72 | Law et al. | Law et al. |
|  | rs3801081 | 7 | 47511161 | G | A | 0.69 | 0.076 | 0.016 | 1.37E-06 | 23.31 | Law et al. | Law et al. |
|  | rs10951878 | 7 | 46926695 | C | T | 0.52 | -0.049 | 0.015 | 8.20E-04 | 11.19 | Law et al. | Law et al. |
|  | rs1412834 | 9 | 22110131 | T | C | 0.49 | -0.104 | 0.015 | 1.05E-12 | 50.74 | Law et al. | Law et al. |
|  | rs7894531 | 10 | 8734761 | G | A | 0.31 | -0.139 | 0.016 | 1.36E-18 | 77.44 | Law et al. | Law et al. |
|  | rs2193352 | 10 | 101346609 | G | A | 0.19 | 0.131 | 0.018 | 7.80E-13 | 51.33 | Law et al. | Law et al. |
|  | rs4450168 | 11 | 10286755 | C | A | 0.16 | 0.107 | 0.024 | 9.26E-06 | 19.66 | Law et al. | Law et al. |
|  | rs4944940 | 11 | 74415252 | G | A | 0.04 | -0.250 | 0.039 | 9.05E-11 | 42.01 | Law et al. | Law et al. |
|  | rs3087967 | 11 | 111156836 | T | C | 0.71 | -0.137 | 0.016 | 1.41E-17 | 72.82 | Law et al. | Law et al. |
|  | rs10849438 | 12 | 6412036 | G | T | 0.12 | 0.093 | 0.024 | 1.02E-04 | 15.09 | Law et al. | Law et al. |
|  | rs45597035 | 13 | 73649152 | A | G | 0.38 | -0.077 | 0.015 | 6.08E-07 | 24.89 | Law et al. | Law et al. |
|  | rs7993934 | 13 | 111074915 | T | C | 0.65 | 0.084 | 0.015 | 5.42E-08 | 29.56 | Law et al. | Law et al. |
|  | rs10161980 | 13 | 34093518 | C | G | 0.37 | -0.088 | 0.015 | 7.46E-09 | 33.41 | Law et al. | Law et al. |
|  | rs16969681 | 15 | 32993111 | T | C | 0.09 | 0.183 | 0.025 | 1.10E-13 | 55.17 | Law et al. | Law et al. |
|  | rs17816465 | 15 | 33156386 | A | G | 0.20 | 0.096 | 0.018 | 1.74E-07 | 27.30 | Law et al. | Law et al. |
|  | rs4776316 | 15 | 67007813 | A | G | 0.27 | -0.085 | 0.018 | 1.42E-06 | 23.26 | Law et al. | Law et al. |
|  | rs7495132 | 15 | 91172901 | T | C | 0.12 | 0.122 | 0.023 | 6.33E-08 | 29.26 | Law et al. | Law et al. |
|  | rs10152518 | 15 | 68177162 | G | A | 0.80 | -0.086 | 0.019 | 3.85E-06 | 21.33 | Law et al. | Law et al. |
|  | rs61336918 | 16 | 80007266 | A | T | 0.71 | -0.091 | 0.017 | 7.54E-08 | 28.92 | Law et al. | Law et al. |
|  | rs899244 | 16 | 86700030 | T | C | 0.22 | 0.091 | 0.018 | 2.77E-07 | 26.40 | Law et al. | Law et al. |
|  | rs9797885 | 19 | 41873001 | G | A | 0.70 | 0.074 | 0.016 | 3.94E-06 | 21.29 | Law et al. | Law et al. |
|  | rs12979278 | 19 | 49218602 | T | C | 0.51 | 0.057 | 0.015 | 1.17E-04 | 14.84 | Law et al. | Law et al. |
|  | rs6085661 | 20 | 6693128 | T | C | 0.38 | 0.094 | 0.015 | 3.11E-10 | 39.60 | Law et al. | Law et al. |
|  | rs2179593 | 20 | 42660286 | A | C | 0.72 | 0.068 | 0.016 | 3.57E-05 | 17.09 | Law et al. | Law et al. |
|  | rs6066825 | 20 | 47340117 | A | G | 0.34 | -0.086 | 0.015 | 2.75E-08 | 30.88 | Law et al. | Law et al. |
|  | rs4811050 | 20 | 48980670 | A | G | 0.18 | 0.120 | 0.019 | 2.32E-10 | 40.17 | Law et al. | Law et al. |
|  | rs6091213 | 20 | 49384745 | C | T | 0.26 | 0.084 | 0.017 | 4.85E-07 | 25.32 | Law et al. | Law et al. |
|  | rs1741640 | 20 | 60932414 | C | T | 0.79 | 0.178 | 0.020 | 2.31E-19 | 80.95 | Law et al. | Law et al. |
|  | rs3787089 | 20 | 62316630 | C | T | 0.69 | -0.065 | 0.017 | 1.34E-04 | 14.58 | Law et al. | Law et al. |

R^2^ of CRC was 9.75%.

SNP, single nucleotide polymorphism; EA, effect allele; OA, other allele; Beta, the estimate of the genetic association between the instrument and the exposure (i.e., log odds ratios for the risk of CRC); EAF, effect allele frequency; SE, standard error.
