# Supplementary material for: Exploring the Complex Relationship between Gut Microbiota and Risk of Colorectal Neoplasia Using Bidirectional Mendelian Randomization Analysis
Source: Cancer Epidemiol Biomarkers Prev. 2023 Apr 3;32(6):809–17. doi: 10.1158/1055-9965.EPI-22-0724 (PMC10233354; doi:10.1158/1055-9965.EPI-22-0724)
Supplement: Table S5 — shows the SNPs for adenoma and polyps. [file epi-22-0724_table_s5_suppst5.docx]

| **Table S5. The SNPs for adenoma and polyps.** | | | | | | | | | | | |
| --- | --- | --- | --- | --- | --- | --- | --- | --- | --- | --- | --- |
| Traits | SNP | Chr | Position | EA | OA | MAF | Beta | SE | P | F-statistics | Reference |
| Adenoma ^a^ | rs11886781 | 2 | 18154780 | C | A | 0.08 | 0.811 | 0.187 | 9.70E-06 | 18.88 | Wang et al |
|  | rs13085889 | 3 | 135843760 | C | A | 0.29 | 0.571 | 0.131 | 8.80E-06 | 19.07 | Wang et al |
|  | rs1381392 | 3 | 28724318 | G | A | 0.18 | 0.698 | 0.142 | 7.40E-07 | 24.28 | Wang et al |
|  | rs17651822 | 3 | 28695130 | G | A | 0.14 | 0.770 | 0.151 | 2.10E-07 | 26.14 | Wang et al |
|  | rs17781398 | 7 | 30807966 | A | G | 0.10 | 1.661 | 0.429 | 9.00E-06 | 15.03 | Wang et al |
|  | rs1424593 | 7 | 131605541 | C | A | 0.50 | 0.580 | 0.129 | 9.10E-06 | 20.30 | Wang et al |
|  | rs1364512 | 7 | 131602384 | C | A | 0.49 | 0.580 | 0.134 | 8.60E-06 | 18.80 | Wang et al |
|  | rs7778725 | 7 | 131614936 | G | A | 0.49 | 0.598 | 0.134 | 4.00E-06 | 19.99 | Wang et al |
|  | rs16909065 | 9 | 121597606 | G | A | 0.05 | 0.952 | 0.212 | 3.60E-06 | 20.25 | Wang et al |
|  | rs16909036 | 9 | 121587049 | A | G | 0.05 | 0.952 | 0.212 | 3.70E-06 | 20.25 | Wang et al |
|  | rs1535989 | 13 | 104820723 | A | G | 0.11 | 0.737 | 0.169 | 8.90E-06 | 19.09 | Wang et al |
|  | rs17654765 | 13 | 104828038 | G | A | 0.10 | 0.761 | 0.170 | 4.70E-06 | 20.12 | Wang et al |
|  | rs9582958 | 13 | 104829133 | A | C | 0.11 | 0.718 | 0.165 | 9.30E-06 | 19.01 | Wang et al |
|  | rs2837156 | 21 | 40048557 | A | G | 0.12 | 0.798 | 0.160 | 3.20E-07 | 25.00 | Wang et al |
|  | rs7278863 | 21 | 40087578 | G | A | 0.10 | 0.908 | 0.164 | 1.40E-08 | 30.85 | Wang et al |
|  | rs2837237 | 21 | 40119727 | A | G | 0.12 | 0.908 | 0.158 | 3.60E-09 | 33.26 | Wang et al |
|  | rs2837241 | 21 | 40130476 | C | A | 0.12 | 0.908 | 0.158 | 3.70E-09 | 33.26 | Wang et al |
|  | rs2837254 | 21 | 40143171 | G | A | 0.11 | 0.936 | 0.162 | 2.90E-09 | 33.62 | Wang et al |
|  | rs741864 | 21 | 40129665 | G | A | 0.11 | 0.908 | 0.163 | 1.10E-08 | 31.23 | Wang et al |
| Polyps ^b^ | rs10505477 | 8 | 128407443 | G | A | 0.50 | 0.073 | 0.047 | 3.00E-03 | 2.41 | Zhang et al |
|  | rs6983267 | 8 | 128413305 | G | T | 0.51 | 0.039 | 0.042 | 1.20E-02 | 0.86 | Zhang et al |
|  | rs7837328 | 8 | 128423127 | A | G | 0.41 | 0.122 | 0.041 | 2.30E-02 | 8.88 | Zhang et al |
|  | rs4779584 | 15 | 32994756 | T | C | 0.18 | 0.058 | 0.041 | 4.10E-04 | 2.00 | Zhang et al |
|  | rs4939827 | 18 | 46453463 | C | T | 0.48 | 0.049 | 0.075 | 1.40E-02 | 0.43 | Zhang et al |
|  | rs10411210 | 19 | 33532300 | T | C | 0.11 | 0.039 | 0.044 | 2.40E-02 | 0.78 | Zhang et al |
|  | rs4925386 | 20 | 60921044 | T | C | 0.30 | 0.077 | 0.050 | 2.00E-03 | 2.37 | Zhang et al |
| ^a^ R^2^ of adenoma was 10.34%. | | | | | | | | | | | |
| ^b^ R^2^ of polyps was 0.72%. | | | | | | | | | | | |

SNP, single nucleotide polymorphism; EA, effect allele; OA, other allele; Beta, the estimate of the genetic association between the instrument and the exposure (i.e., the risk of adenoma and polyps); MAF, minor allele frequency; SE, standard error.
