# Supplementary material for: Exploring the Complex Relationship between Gut Microbiota and Risk of Colorectal Neoplasia Using Bidirectional Mendelian Randomization Analysis
Source: Cancer Epidemiol Biomarkers Prev. 2023 Apr 3;32(6):809–17. doi: 10.1158/1055-9965.EPI-22-0724 (PMC10233354; doi:10.1158/1055-9965.EPI-22-0724)
Supplement: Table S6 — shows the forward MR analyses of 24 gut microbiota with the risk of colorectal cancer. [file epi-22-0724_table_s6_suppst6.docx]

**Table S6. The forward MR analyses of 24 gut microbiota with the risk of colorectal cancer.**

| Traits | Method | N_SNP_ | OR (95%CI) | P for effect | FDR |
| --- | --- | --- | --- | --- | --- |
| *Actinobacteria ^1^* | Wald ratio | 1 | 0.94(0.58, 1.53) | 0.815 | 0.954 |
| *Bifidobacteriaceae* | IVW | 2 | 0.88(0.61, 1.28) | 0.502 | 0.948 |
| *Oxalobacteraceae** | - | - | - | - | - |
| *Peptostreptococcaceae* | Wald ratio | 1 | 1.27(0.83, 1.96) | 0.277 | 0.948 |
| *Streptococcaceae* | Wald ratio | 1 | 1.36(0.84, 2.19) | 0.207 | 0.948 |
| *Eubacterium coprostanoligenes group* | Wald ratio | 1 | 1.06(0.57, 1.95) | 0.860 | 0.954 |
| *Ruminococcus torques group** | - | - | - | - | - |
| *Allisonella* | Wald ratio | 1 | 1.01(0.83, 1.22) | 0.954 | 0.954 |
| *Bifidobacterium* | IVW | 2 | 0.89(0.63, 1.26) | 0.512 | 0.948 |
| *Enterorhabdus* | Wald ratio | 1 | 0.75(0.55, 1.01) | 0.062 | 0.600 |
| *Erysipelatoclostridium* | Wald ratio | 1 | 1.02(0.71, 1.45) | 0.930 | 0.954 |
| *Faecalibacterium** | - | - | - | - | - |
| *Intestinibacter* | Wald ratio | 1 | 0.88(0.59, 1.32) | 0.550 | 0.948 |
| *Oxalobacter* | Wald ratio | 1 | 1.01(0.79, 1.30) | 0.931 | 0.954 |
| *Peptococcus** | - | - | - | - | - |
| *Romboutsia* | Wald ratio | 1 | 1.27(0.83, 1.94) | 0.277 | 0.948 |
| *RuminococcaceaeUCG013* | Wald ratio | 1 | 1.76(1.10, 2.80) | 0.018 | 0.228 |
| *RuminococcaceaeUCG009** | - | - | - | - | - |
| *Ruminococcus1* | Wald ratio | 1 | 1.12(0.70, 1.81) | 0.632 | 0.954 |
| *Streptococcus* | Wald ratio | 1 | 1.33(0.84, 2.12) | 0.229 | 0.948 |
| *Tyzzerella3* | Wald ratio | 1 | 1.14(0.87, 1.51) | 0.340 | 0.948 |
| *Bifidobacteriales* | IVW | 2 | 0.88(0.61, 1.28) | 0.502 | 0.948 |
| *Gastranaerophilales* | Wald ratio | 1 | 1.09(0.82, 1.44) | 0.559 | 0.948 |
| *Actinobacteria ^2^* | Wald ratio | 1 | 0.91(0.55, 1.49) | 0.696 | 0.954 |

MR, mendelian randomization; OR, odds ratio, which represents the risk in colorectal cancer with each log-transformed higher abundance in each gut microbial trait; CI, confidence interval; FDR, false discovery rate; IVW, inverse variance weighted.

* The SNPs for the five gut microbiota taxa are excluded in the process of harmonization

^1^ class *Actinobacteria*

**^2^** phylum *Actinobacteria*
