# Supplementary material for: Exploring the Complex Relationship between Gut Microbiota and Risk of Colorectal Neoplasia Using Bidirectional Mendelian Randomization Analysis
Source: Cancer Epidemiol Biomarkers Prev. 2023 Apr 3;32(6):809–17. doi: 10.1158/1055-9965.EPI-22-0724 (PMC10233354; doi:10.1158/1055-9965.EPI-22-0724)
Supplement: Table S7 — shows the forward MR analyses of nine gut microbiota with the risk of colorectal cancer. [file epi-22-0724_table_s7_suppst7.docx]

**Table S7. The forward MR analyses of nine gut microbiota with the risk of colorectal cancer.**

| Trait | IVW | | | | Weighted Median | | Weighted Mode | | MR-Egger | | | MR-PRESSO | | |
| --- | --- | --- | --- | --- | --- | --- | --- | --- | --- | --- | --- | --- | --- | --- |
|  | OR (95%CI) | P for effect | FDR | P for heterogeneity | OR (95%CI) | P for effect | OR (95%CI) | P for effect | OR (95%CI) | P for effect | P for pleiotropy. | OR (95%CI) | P for effect | P for Global Test |
| *Gammaproteobacteria* | 0.99(0.74, 1.32) | 0.933 | 0.954 | 0.149 | 1.13(0.83, 1.55) | 0.432 | 1.15(0.78, 1.69) | 0.508 | 0.84(0.32, 2.21) | 0.736 | 0.739 | 0.97(0.93,1.01) | 0.463 | 0.411 |
| *Lactobacillales* | 0.99(0.86, 1.15) | 0.940 | 0.954 | 0.730 | 1.03(0.85, 1.26) | 0.742 | 1.27(0.87, 1.85) | 0.228 | 1.68(1.11, 2.53) | 0.025 | 0.017 | 0.99(0.91,1.05) | 0.758 | 0.743 |
| *Enterobacteriaceae* | 1.06(0.89, 1.26) | 0.505 | 0.948 | 0.219 | 1.07(0.85, 1.34) | 0.567 | 1.04(0.73, 1.48) | 0.837 | 0.83(0.37, 1.89) | 0.674 | 0.572 | 1.04(0.88,1.08) | 0.105 | 0.736 |
| *Porphyromonadaceae* | 1.06(0.89, 1.27) | 0.517 | 0.948 | 0.443 | 1.02(0.79, 1.31) | 0.892 | 0.94(0.65, 1.38) | 0.771 | 0.73(0.34, 1.59) | 0.452 | 0.360 | 0.89(0.74,1.01) | 0.568 | 0.249 |
| *Fusobacteriaceae* | 1.03(0.80, 1.33) | 0.803 | 0.954 | 0.599 | 1.00(0.68, 1.46) | 0.999 | 1.08(0.6, 1.98) | 0.794 | 1.10(0.67, 1.8) | 0.699 | 0.761 | 1.03(0.81, 1.32) | 0.795 | 0.528 |
| *Actinomyces* | 0.91(0.61, 1.37) | 0.668 | 0.954 | 0.344 | 0.94(0.67, 1.32) | 0.728 | 0.98(0.62, 1.53) | 0.928 | 0.49(0.03, 7.23) | 0.695 | 0.724 | 0.91(0.88,1.03) | 0.177 | 0.114 |
| *Bifidobacterium* | 0.96(0.83, 1.10) | 0.523 | 0.948 | 0.228 | 0.99(0.82, 1.19) | 0.904 | 0.98(0.75, 1.29) | 0.893 | 0.89(0.58, 1.35) | 0.577 | 0.707 | 0.98(0.97,1.02) | 0.464 | 0.272 |
| *Roseburia* | 0.91(0.72, 1.14) | 0.412 | 0.948 | 0.001 | 0.84(0.67, 1.07) | 0.155 | 0.76(0.44, 1.32) | 0.348 | 1.28(0.54, 3.04) | 0.585 | 0.434 | 0.92(0.88,1.13) | 0.103 | 0.068 |
| *Peptostreptococcaceae* | 1.02(0.84, 1.15) | 0.832 | 0.948 | 0.295 | 0.97(0.81, 1.20) | 0.793 | 0.98(0.77, 1.26) | 0.863 | 0.93(0.69, 1.40) | 0.676 | 0.570 | 0.98(0.86, 1.13) | 0.814 | 0.391 |

MR, mendelian randomization; OR, odds ratio, which represents the risk in colorectal cancer with each log-transformed higher abundance in each gut microbial trait; CI, confidence interval; FDR, false discovery rate; IVW, inverse variance weighted; MR-PRESSO, MR pleiotropy residual sum and outlier test.
