# Supplementary material for: Exploring the Complex Relationship between Gut Microbiota and Risk of Colorectal Neoplasia Using Bidirectional Mendelian Randomization Analysis
Source: Cancer Epidemiol Biomarkers Prev. 2023 Apr 3;32(6):809–17. doi: 10.1158/1055-9965.EPI-22-0724 (PMC10233354; doi:10.1158/1055-9965.EPI-22-0724)
Supplement: Table S8 — shows the forward MR analyses of the six gut microbial metabolites with the risk of colorectal cancer. [file epi-22-0724_table_s8_suppst8.docx]

**Table S8. The forward MR analyses of the six gut microbial metabolites with the risk of colorectal cancer**

| Metabolites | IVW | | | | Weighted Median | | Weighted Mode | | MR-Egger | | | MR-PRESSO | | |
| --- | --- | --- | --- | --- | --- | --- | --- | --- | --- | --- | --- | --- | --- | --- |
|  | OR (95%CI) | P for effect | FDR | P for heterogeneity | OR (95%CI) | P for effect | OR (95%CI) | P for effect | OR (95%CI) | P for effect | P for pleiotropy | OR (95%CI) | P for effect | P for Global Test |
| Choline | 0.99(0.89, 1.10) | 0.862 | 0.954 | 0.054 | 1.00(0.90, 1.11) | 0.971 | 1.03(0.86, 1.22) | 0.783 | 1.03(0.72, 1.47) | 0.880 | 0.832 | 1.04(0.98, 1.65) | 0.310 | 0.532 |
| Betaine | 0.98(0.92, 1.05) | 0.616 | 0.954 | 0.051 | 1.03(0.95, 1.11) | 0.506 | 1.05(0.95, 1.14) | 0.361 | 0.95(0.80, 1.14) | 0.607 | 0.715 | 0.89(0.76, 1.23) | 0.261 | 0.121 |
| Carnitine | 0.98(0.92, 1.04) | 0.526 | 0.948 | 0.107 | 0.95(0.89, 1.01) | 0.101 | 0.95(0.87, 1.03) | 0.252 | 0.93(0.74, 1.16) | 0.533 | 0.629 | 0.99(0.97, 1.04) | 0.732 | 0.152 |
| TMAO | 0.99(0.92, 1.06) | 0.697 | 0.954 | 0.576 | 0.96(0.89, 1.03) | 0.266 | 0.96(0.87, 1.05) | 0.375 | 0.86(0.68, 1.09) | 0.261 | 0.284 | 0.99(0.92, 1.06) | 0.708 | 0.255 |
| GABA | 0.97(0.94, 1.00) | 0.093 | 0.725 | 0.995 | 0.97(0.93, 1.01) | 0.164 | 0.97(0.93, 1.01) | 0.194 | 0.96(0.91, 1.02) | 0.215 | 0.707 | 1.12(0.85, 1.13) | 0.883 | 0.184 |
| Propionic acid | 0.98(0.87, 1.12) | 0.806 | 0.954 | 0.080 | - | - | - | - | - | - | - | - | - | - |

MR, mendelian randomization; TMAO, trimethylamine N-oxide; GAMA, gamma-aminobutyric acid; OR, odds ratio, which represents the risk in colorectal cancer with each log-transformed higher concentrations (i.e., μM) in each gut microbial metabolites; CI, confidence interval; FDR, false discovery rate; IVW, inverse variance weighted; MR-PRESSO, MR pleiotropy residual sum and outlier test.
