# Supplementary material for: Exploring the Complex Relationship between Gut Microbiota and Risk of Colorectal Neoplasia Using Bidirectional Mendelian Randomization Analysis
Source: Cancer Epidemiol Biomarkers Prev. 2023 Apr 3;32(6):809–17. doi: 10.1158/1055-9965.EPI-22-0724 (PMC10233354; doi:10.1158/1055-9965.EPI-22-0724)
Supplement: Table S9 — shows the reverse MR analyses of colorectal cancer, adenoma and polyps with the abundance of nine gut microbiota. [file epi-22-0724_table_s9_suppst9.docx]

**Table S9. The reverse MR analyses of colorectal cancer, adenoma and polyps with the abundance of nine gut microbiota**

| Trait | IVW | | | | Weighted median | | Weighted mode | | MR-Egger | | | MR-PRESSO | | |
| --- | --- | --- | --- | --- | --- | --- | --- | --- | --- | --- | --- | --- | --- | --- |
|  | β(95%CI) | P for effect | FDR | P for heterogeneity | β(95%CI) | P for effect | β(95%CI) | P for effect | β(95%CI) | P for effect | P for pleiotropy | β(95%CI) | P for effect | P for Global Test |
| CRC |  |  |  |  |  |  |  |  |  |  |  |  |  |  |
| *Gammaproteobacteria* | 0.005(-0.032, 0.042) | 0.801 | 0.973 | 0.005 | -0.009(-0.061, 0.043) | 0.747 | -0.014(-0.118, 0.09) | 0.794 | 0.014(-0.108, 0.136) | 0.821 | 0.875 | 0.008(-0.015, 0.031) | 0.012 | 0.148 |
| *Lactobacillales* | -0.006(-0.037, 0.025) | 0.731 | 0.973 | 0.400 | -0.005(-0.053, 0.043) | 0.828 | 0.040(-0.005, 0.013) | 0.388 | -0.079(-0.185, 0.027) | 0.145 | 0.155 | -0.001(-0.014, 0.015) | 0.965 | 0.215 |
| *Enterobacteriaceae* | 0.004(-0.039, 0.031) | 0.811 | 0.973 | 0.632 | 0.009(-0.047, 0.065) | 0.740 | 0.019(-0.091, 0.129) | 0.731 | 0.051(-0.067, 0.169) | 0.395 | 0.543 | 0.017(0.001, 0.033) | 0.040 | 0.368 |
| *Porphyromonadaceae* | 0.0003(-0.031, 0.032) | 0.983 | 0.998 | 0.457 | 0.022(-0.059, 0.103) | 0.594 | 0.022(-0.058, 0.102) | 0.594 | -0.038(-0.14, 0.064) | 0.466 | 0.440 | 0.002(-0.011, 0.015) | 0.794 | 0.546 |
| *Fusobacteriaceae* | 0.014(-0.002, 0.03) | 0.089 | 0.695 | 0.987 | 0.017(-0.007, 0.041) | 0.166 | 0.003(-0.034, 0.040) | 0.881 | -0.002(-0.051, 0.047) | 0.946 | 0.502 | -0.019(-0.051, 0.012) | 0.237 | 0.753 |
| *Actinomyces* | -0.021(-0.070, 0.028) | 0.392 | 0.973 | 0.710 | -0.039(-0.115, 0.037) | 0.317 | -0.025(-0.156, 0.106) | 0.713 | 0.025(-0.134, 0.184) | 0.760 | 0.555 | 0.008(-0.011, 0.027) | 0.424 | 0.868 |
| *Bifidobacterium* | 0.008(-0.027, 0.043) | 0.654 | 0.973 | 0.707 | -0.026(-0.077, 0.025) | 0.314 | -0.042(-0.124, 0.040) | 0.323 | -0.109(-0.221, 0.003) | 0.062 | 0.037 | 0.001(-0.016, 0.015) | 0.982 | 0.307 |
| *Roseburia* | 0.032(-0.009, 0.073) | 0.129 | 0.815 | 0.129 | 0.019(-0.033, 0.071) | 0.471 | 0.001(-0.101, 0.103) | 0.977 | -0.068(-0.201, 0.065) | 0.323 | 0.129 | -0.005(-0.022, 0.011) | 0.528 | 0.002 |
| *Peptostreptococcaceae* | -0.004(-0.039, 0.031) | 0.819 | 0.973 | 0.172 | 0.059(0.008, 0.11) | 0.026 | 0.084(-0.002, 0.17) | 0.061 | 0.112(0.018, 0.206) | 0.022 | 0.011 | 0.002(-0.031, 0.035) | 0.890 | 0.163 |
| *Actinobacteria^1^* | -0.007(-0.04, 0.026) | 0.664 | 0.973 | 0.426 | -0.003(-0.054, 0.048) | 0.897 | 0.018(-0.066, 0.102) | 0.665 | 0.005(-0.087, 0.097) | 0.914 | 0.777 | -0.003(-0.034, 0.028) | 0.866 | 0.437 |
| *Bifidobacteriaceae* | 0.006(-0.029, 0.041) | 0.730 | 0.973 | 0.233 | 0.027(-0.026, 0.08) | 0.320 | 0.036(-0.042, 0.114) | 0.372 | 0.029(-0.071, 0.129) | 0.571 | 0.635 | 0.008(-0.027, 0.043) | 0.664 | 0.201 |
| *Oxalobacteraceae* | -0.057(-0.12, 0.006) | 0.075 | 0.642 | 0.121 | -0.017(-0.109, 0.075) | 0.719 | -0.014(-0.163, 0.135) | 0.849 | 0.025(-0.149, 0.199) | 0.777 | 0.323 | -0.060(-0.123, 0.003) | 0.065 | 0.040 |
| *Streptococcaceae* | -0.006(-0.039, 0.027) | 0.720 | 0.973 | 0.468 | -0.006(-0.055, 0.043) | 0.815 | -0.012(-0.086, 0.062) | 0.755 | -0.011(-0.101, 0.079) | 0.810 | 0.905 | -0.001(-0.033, 0.033) | 0.986 | 0.321 |
| *Eubacterium coprostanoligenes group* | -0.011(-0.042, 0.02) | 0.512 | 0.973 | 0.981 | -0.015(-0.062, 0.032) | 0.534 | -0.013(-0.087, 0.061) | 0.737 | 0.004(-0.084, 0.092) | 0.921 | 0.718 | -0.006(-0.031, 0.019) | 0.669 | 0.986 |
| *Ruminococcus torques group* | -0.019(-0.056, 0.018) | 0.314 | 0.973 | 0.009 | -0.026(-0.073, 0.021) | 0.280 | -0.03(-0.101, 0.041) | 0.418 | -0.018(-0.122, 0.086) | 0.730 | 0.986 | -0.017(-0.052, 0.018) | 0.350 | 0.013 |
| *Allisonella* | -0.071(-0.149, 0.007) | 0.074 | 0.642 | 0.896 | -0.076(-0.19, 0.038) | 0.189 | -0.079(-0.271, 0.113) | 0.421 | 0.034(-0.18, 0.248) | 0.756 | 0.306 | -0.047(-0.116, 0.022) | 0.178 | 0.869 |
| *Enterorhabdus* | 0.024(-0.025, 0.073) | 0.345 | 0.973 | 0.277 | 0.047(-0.029, 0.123) | 0.222 | 0.104(-0.033, 0.241) | 0.142 | 0.106(-0.031, 0.243) | 0.135 | 0.214 | 0.027(-0.02, 0.074) | 0.256 | 0.387 |
| *Erysipelatoclostridium* | -0.012(-0.057, 0.033) | 0.590 | 0.973 | 0.219 | 0.007(-0.06, 0.074) | 0.838 | 0.021(-0.087, 0.129) | 0.704 | -0.019(-0.142, 0.104) | 0.759 | 0.903 | -0.01(-0.053, 0.033) | 0.636 | 0.275 |
| *Faecalibacterium* | -0.016(-0.047, 0.015) | 0.330 | 0.973 | 0.319 | -0.022(-0.073, 0.029) | 0.388 | -0.051(-0.145, 0.043) | 0.294 | 0.029(-0.059, 0.117) | 0.524 | 0.290 | -0.014(-0.045, 0.017) | 0.373 | 0.242 |
| *Intestinibacter* | 0.002(-0.035, 0.039) | 0.908 | 0.973 | 0.523 | 0.011(-0.046, 0.068) | 0.700 | 0.04(-0.058, 0.138) | 0.421 | 0.101(-0.003, 0.205) | 0.060 | 0.049 | 0.010(-0.025, 0.045) | 0.584 | 0.576 |
| *Oxalobacter* | -0.050(-0.113, 0.013) | 0.118 | 0.815 | 0.366 | -0.064(-0.16, 0.032) | 0.192 | -0.099(-0.268, 0.07) | 0.253 | 0.016(-0.16, 0.192) | 0.857 | 0.433 | -0.053(-0.116, 0.01) | 0.098 | 0.227 |
| *Peptococcus* | 0.011(-0.048, 0.07) | 0.722 | 0.973 | 0.150 | 0.021(-0.065, 0.107) | 0.628 | 0.01(-0.131, 0.151) | 0.885 | -0.04(-0.207, 0.127) | 0.641 | 0.524 | 0.022(-0.037, 0.081) | 0.460 | 0.088 |
| *Peptococcus* | -0.018(-0.079, 0.043) | 0.557 | 0.973 | 0.112 | -0.028(-0.114, 0.058) | 0.528 | -0.022(-0.173, 0.129) | 0.777 | -0.019(-0.221, 0.183) | 0.854 | 0.993 | -0.006(-0.032, 0.019) | 0.632 | 0.149 |
| *Romboutsia* | -0.004(-0.039, 0.031) | 0.834 | 0.973 | 0.576 | 0.017(-0.036, 0.07) | 0.521 | 0.09(-0.01, 0.19) | 0.078 | 0.083(-0.013, 0.179) | 0.096 | 0.062 | -0.005(-0.038, 0.028) | 0.769 | 0.595 |
| *RuminococcaceaeUCG013* | -0.013(-0.048, 0.022) | 0.472 | 0.973 | 0.071 | 0.006(-0.043, 0.055) | 0.808 | 0.007(-0.062, 0.076) | 0.836 | -0.01(-0.110, 0.090) | 0.847 | 0.945 | -0.005(-0.040, 0.030) | 0.779 | 0.081 |
| *RuminococcaceaeUCG009* | -0.009(-0.06, 0.042) | 0.725 | 0.973 | 0.149 | -0.012(-0.088, 0.064) | 0.755 | -0.013(-0.127, 0.101) | 0.821 | -0.018(-0.163, 0.127) | 0.812 | 0.905 | -0.027(-0.078, 0.024) | 0.290 | 0.124 |
| *Ruminococcus1* | -0.006(-0.039, 0.027) | 0.750 | 0.973 | 0.263 | 0.022(-0.027, 0.071) | 0.368 | 0.026(-0.052, 0.104) | 0.513 | 0.057(-0.035, 0.149) | 0.231 | 0.160 | -0.002(-0.035, 0.031) | 0.895 | 0.211 |
| *Streptococcus* | -0.006(-0.039, 0.027) | 0.703 | 0.973 | 0.552 | -0.014(-0.065, 0.037) | 0.584 | -0.014(-0.088, 0.06) | 0.713 | 0.001(-0.089, 0.091) | 0.981 | 0.862 | -0.001(-0.032, 0.03) | 0.929 | 0.487 |
| *Tyzzerella3* | -0.066(-0.123, -0.009) | 0.024 | 0.375 | 0.932 | -0.081(-0.169, 0.007) | 0.072 | -0.107(-0.262, 0.048) | 0.180 | -0.227(-0.386, -0.068) | 0.006 | 0.035 | -0.072(-0.123, -0.021) | 0.007 | 0.855 |
| *Gastranaerophilales* | -0.030(-0.085, 0.025) | 0.282 | 0.973 | 0.766 | -0.011(-0.091, 0.069) | 0.788 | -0.016(-0.138, 0.106) | 0.801 | 0.062(-0.089, 0.213) | 0.425 | 0.205 | -0.032(-0.081, 0.017) | 0.206 | 0.784 |
| *Actinobacteria^2^* | -0.002(-0.033, 0.029) | 0.895 | 0.973 | 0.773 | -0.007(-0.052, 0.038) | 0.781 | 0.011(-0.058, 0.08) | 0.759 | -0.009(-0.095, 0.077) | 0.832 | 0.860 | 0.006(-0.023, 0.035) | 0.691 | 0.732 |
| Adenoma |  |  |  |  |  |  |  |  |  |  |  |  |  |  |
| *Gammaproteobacteria* | 0.027(0.017, 0.037) | 7.06E-08 | 8.83E-06 | 0.413 | 0.029(0.015, 0.043) | 4.04E-05 | 0.031(0.011, 0.052) | 0.010 | 0.049(0.013, 0.085) | 0.013 | 0.232 | 0.027(0.017, 0.037) | 4.89×10^-5^ | 0.535 |
| *Lactobacillales* | 0.004(-0.005, 0.014) | 0.349 | 0.973 | 0.764 | 0.003(-0.010, 0.016) | 0.662 | -0.016(-0.036, 0.004) | 0.143 | 0.024(-0.010, 0.058) | 0.193 | 0.268 | 0.004(-0.004, 0.012) | 0.292 | 0.791 |
| *Enterobacteriaceae* | 0.023(0.013, 0.034) | 1.29E-05 | 8.06E-04 | 0.466 | 0.018(0.002, 0.034) | 0.032 | 0.015(-0.010, 0.040) | 0.263 | 0.050(0.011, 0.089) | 0.023 | 0.185 | 0.023(0.013, 0.034) | 0.001 | 0.536 |
| *Porphyromonadaceae* | -0.003(-0.012, 0.006) | 0.536 | 0.973 | 0.872 | -0.007(-0.019, 0.005) | 0.239 | 0.009(-0.008, 0.026) | 0.335 | -0.020(-0.054, 0.013) | 0.251 | 0.304 | -0.003(-0.010, 0.004) | 0.446 | 0.867 |
| *Fusobacteriaceae* | 0.006(0.002, 0.011) | 0.006 | 0.188 | 0.722 | 0.004(-0.002, 0.010) | 0.174 | 0.008(-0.003, 0.018) | 0.168 | -0.001(-0.019, 0.017) | 0.917 | 0.423 | 0.006(0.002, 0.010) | 0.006 | 0.730 |
| *Actinomyces* | 0.002(-0.012, 0.02) | 0.798 | 0.973 | 0.929 | 0.007(-0.013, 0.026) | 0.514 | -0.018(-0.053, 0.017) | 0.321 | -0.012(-0.064, 0.040) | 0.661 | 0.598 | 0.002(-0.009, 0.012) | 0.731 | 0.935 |
| *Bifidobacterium* | -0.001(-0.011, 0.009) | 0.861 | 0.973 | 0.963 | -0.002(-0.015, 0.011) | 0.748 | 0.001(-0.018, 0.020) | 0.924 | -0.008(-0.045, 0.029) | 0.672 | 0.695 | -0.001(-0.008, 0.006) | 0.803 | 0.966 |
| *Roseburia* | -0.008(-0.0018, 0.001) | 0.073 | 0.642 | 0.949 | -0.010(-0.022, 0.003) | 0.134 | -0.047(-0.102, 0.008) | 0.113 | 0.019(-0.014, 0.053) | 0.280 | 0.114 | -0.008(-0.015, -0.002) | 0.023 | 0.959 |
| *Peptostreptococcaceae* | 0.002(-0.008, 0.012) | 0.694 | 0.973 | 0.931 | 0.003(-0.011, 0.017) | 0.667 | 0.011(-0.013, 0.035) | 0.348 | 0.034(-0.001, 0.069) | 0.079 | 0.085 | 0.002(-0.006, 0.01) | 0.601 | 0.933 |
| *Actinobacteria^1^* | -0.002(-0.012, 0.008) | 0.698 | 0.973 | 0.978 | -0.003(-0.017, 0.011) | 0.642 | -0.003(-0.021, 0.015) | 0.732 | 0.002(-0.033, 0.037) | 0.905 | 0.817 | -0.002(-0.008, 0.004) | 0.563 | 0.981 |
| *Bifidobacteriaceae* | -0.001(-0.010, 0.010) | 0.959 | 0.991 | 0.964 | -0.002(-0.016, 0.012) | 0.767 | -0.002(-0.022, 0.018) | 0.867 | -0.005(-0.042, 0.032) | 0.787 | 0.790 | -0.001(-0.008, 0.008) | 0.942 | 0.971 |
| *Oxalobacteraceae* | -0.018(-0.036, -0.001) | 0.043 | 0.538 | 0.742 | -0.025(-0.049, -0.001) | 0.044 | -0.025(-0.06, 0.010) | 0.172 | -0.05(-0.113, 0.013) | 0.136 | 0.305 | -0.018(-0.034, -0.002) | 0.033 | 0.753 |
| *Streptococcaceae* | 0.004(-0.006, 0.014) | 0.438 | 0.973 | 0.554 | 0.002(-0.012, 0.016) | 0.758 | -0.004(-0.024, 0.016) | 0.739 | 0.023(-0.012, 0.058) | 0.221 | 0.286 | 0.004(-0.006, 0.014) | 0.429 | 0.580 |
| *Eubacterium coprostanoligenes group* | -0.002(-0.012, 0.008) | 0.630 | 0.973 | 0.707 | -0.001(-0.015, 0.013) | 0.932 | 0.008(-0.017, 0.033) | 0.518 | -0.008(-0.041, 0.025) | 0.649 | 0.735 | -0.002(-0.01, 0.006) | 0.595 | 0.708 |
| *Ruminococcus torques group* | 0.007(-0.003, 0.017) | 0.137 | 0.815 | 0.789 | 0.006(-0.006, 0.018) | 0.369 | 0.008(-0.01, 0.026) | 0.425 | 0.002(-0.031, 0.035) | 0.925 | 0.750 | 0.007(-0.001, 0.015) | 0.097 | 0.805 |
| *Allisonella* | 0.04(0.007, 0.073) | 0.021 | 0.375 | 0.078 | 0.039(0.001, 0.078) | 0.049 | 0.053(0.001, 0.106) | 0.074 | 0.222(0.077, 0.367) | 0.013 | 0.031 | 0.04(0.007, 0.073) | 0.041 | 0.105 |
| *Enterorhabdus* | -0.002(-0.022, 0.018) | 0.798 | 0.973 | 0.023 | -0.011(-0.035, 0.013) | 0.331 | -0.029(-0.068, 0.01) | 0.177 | -0.095(-0.148, -0.042) | 0.003 | 0.003 | -0.002(-0.022, 0.018) | 0.801 | 0.017 |
| *Erysipelatoclostridium* | -0.003(-0.015, 0.009) | 0.619 | 0.973 | 0.996 | -0.002(-0.018, 0.014) | 0.818 | -0.002(-0.024, 0.02) | 0.852 | -0.003(-0.048, 0.042) | 0.900 | 0.993 | -0.003(-0.011, 0.005) | 0.394 | 0.995 |
| *Faecalibacterium* | -0.007(-0.017, 0.003) | 0.131 | 0.815 | 0.999 | -0.009(-0.021, 0.003) | 0.131 | -0.01(-0.028, 0.008) | 0.296 | 0.008(-0.025, 0.041) | 0.633 | 0.363 | -0.007(-0.011, -0.003) | 0.007 | 0.999 |
| *Intestinibacter* | -0.006(-0.02, 0.008) | 0.403 | 0.973 | 0.047 | 0.007(-0.011, 0.025) | 0.417 | 0.012(-0.013, 0.037) | 0.371 | 0.053(0.010, 0.096) | 0.031 | 0.015 | -0.006(-0.02, 0.008) | 0.415 | 0.053 |
| *Oxalobacter* | -0.006(-0.026, 0.014) | 0.527 | 0.973 | 0.341 | -0.017(-0.041, 0.007) | 0.178 | -0.02(-0.053, 0.013) | 0.263 | -0.062(-0.129, 0.005) | 0.089 | 0.109 | -0.006(-0.026, 0.014) | 0.537 | 0.405 |
| *Peptococcus* | -0.011(-0.027, 0.005) | 0.202 | 0.973 | 0.924 | -0.017(-0.041, 0.007) | 0.156 | -0.022(-0.055, 0.011) | 0.229 | -0.054(-0.113, 0.005) | 0.096 | 0.159 | -0.011(-0.023, 0.001) | 0.103 | 0.929 |
| *Peptococcus* | -0.011(-0.027, 0.006) | 0.202 | 0.973 | 0.924 | -0.017(-0.039, 0.005) | 0.129 | 0.022(-0.013, 0.056) | 0.232 | -0.054(-0.114, 0.006) | 0.096 | 0.159 | -0.011(-0.023, 0.001) | 0.103 | 0.922 |
| *Romboutsia* | -0.009(-0.019, 0.001) | 0.075 | 0.642 | 0.911 | -0.008(-0.022, 0.006) | 0.273 | -0.003(-0.025, 0.019) | 0.819 | 0.025(-0.012, 0.062) | 0.215 | 0.083 | -0.009(-0.017, -0.001) | 0.032 | 0.919 |
| *RuminococcaceaeUCG013* | 0.014(0.002, 0.026) | 0.018 | 0.375 | 0.110 | 0.015(0.001, 0.029) | 0.049 | 0.015(-0.01, 0.04) | 0.270 | 0.025(-0.018, 0.068) | 0.275 | 0.610 | 0.014(0.002, 0.026) | 0.030 | 0.133 |
| *RuminococcaceaeUCG009* | -0.025(-0.039, -0.011) | 0.001 | 0.052 | 0.399 | -0.017(-0.037, 0.003) | 0.097 | -0.008(-0.041, 0.025) | 0.655 | 0.012(-0.041, 0.065) | 0.660 | 0.171 | -0.025(-0.039, -0.011) | 0.004 | 0.402 |
| *Ruminococcus1* | 0.004(-0.008, 0.016) | 0.521 | 0.973 | 0.052 | 0.008(-0.006, 0.022) | 0.234 | 0.012(-0.006, 0.03) | 0.215 | 0.012(-0.033, 0.057) | 0.619 | 0.731 | 0.004(-0.008, 0.016) | 0.530 | 0.063 |
| *Streptococcus* | 0.004(-0.006, 0.014) | 0.423 | 0.973 | 0.233 | 0.003(-0.011, 0.017) | 0.644 | 0.022(-0.003, 0.047) | 0.099 | 0.027(-0.012, 0.066) | 0.190 | 0.251 | 0.004(-0.006, 0.014) | 0.434 | 0.235 |
| *Tyzzerella3* | 0.007(-0.022, 0.036) | 0.632 | 0.973 | 0.001 | -0.009(-0.038, 0.02) | 0.533 | -0.024(-0.089, 0.041) | 0.476 | -0.011(-0.123, 0.101) | 0.855 | 0.748 | 0.007(-0.022, 0.036) | 0.638 | 0.006 |
| *Gastranaerophilales* | -0.001(-0.016, 0.016) | 0.990 | 0.998 | 0.578 | -0.007(-0.029, 0.015) | 0.499 | -0.016(-0.047, 0.015) | 0.325 | -0.004(-0.063, 0.055) | 0.899 | 0.892 | 0.001(-0.016, 0.016) | 0.989 | 0.618 |
| *Actinobacteria^2^* | 0.006(-0.004, 0.016) | 0.237 | 0.973 | 0.887 | 0.001(-0.011, 0.013) | 0.871 | 0.001(-0.019, 0.021) | 0.957 | -0.027(-0.06, 0.006) | 0.138 | 0.068 | 0.006(-0.002, 0.014) | 0.148 | 0.896 |
| Polyps |  |  |  |  |  |  |  |  |  |  |  |  |  |  |
| *Gammaproteobacteria* | 0.107(-0.138, 0.353) | 0.392 | 0.973 | 0.044 | 0.019(-6.016, 6.055) | 0.044 | 0.016(-0.151, 0.183) | 0.870 | -0.315(-1.471, 0.841) | 0.688 | 0.595 | - | - | - |
| *Lactobacillales* | 0.036(-0.098, 0.170) | 0.599 | 0.973 | 0.401 | 0.046(-0.410, 0.501) | 0.668 | 0.056(-0.115, 0.226) | 0.589 | 0.123(-0.429, 0.676) | 0.738 | 0.803 | - | - | - |
| *Enterobacteriaceae* | 0.109(-0.144, 0.363) | 0.398 | 0.973 | 0.029 | 0.021(-0.726, 0.768) | 0.059 | 0.019(-0.168, 0.206) | 0.858 | -0.183(-1.545, 1.179) | 0.836 | 0.740 | - | - | - |
| *Porphyromonadaceae* | -0.019(-0.150, 0.112) | 0.775 | 0.973 | 0.886 | -0.010(-0.736, 0.717) | 0.385 | 0.055(-0.101, 0.212) | 0.560 | 0.349(-0.192, 0.890) | 0.426 | 0.401 | - | - | - |
| *Fusobacteriaceae* | 0.024(-0.073, 0.122) | 0.627 | 0.973 | 0.586 | - | - | - | - | - | - | - | - | - | - |
| *Actinomyces* | -0.011(-0.214, 0.193) | 0.919 | 0.973 | 0.689 | -0.013(-6.557, 6.530) | 0.418 | -0.118(-0.379, 0.142) | 0.468 | -0.534(-1.374, 0.306) | 0.431 | 0.427 | - | - | - |
| *Bifidobacterium* | -0.032(-0.177, 0.112) | 0.663 | 0.973 | 0.644 | -0.050(-0.338, 0.238) | 0.844 | -0.054(-0.239, 0.132) | 0.628 | -0.137(-0.734, 0.461) | 0.732 | 0.784 | - | - | - |
| *Roseburia* | -0.047(-0.348, 0.253) | 0.758 | 0.973 | 0.195 | -0.103(-0.571, 0.364) | 0.005 | -0.133(-0.320, 0.055) | 0.300 | -0.843(-1.548, -0.139) | 0.257 | 0.263 |  |  |  |
| *Bifidobacterium* | -0.032(-0.177, 0.113) | 0.663 | 0.973 | 0.844 | -0.05(-0.581, 0.481) | 0.854 | -0.054(-0.23, 0.122) | 0.611 | -0.137(-0.735, 0.461) | 0.732 | 0.784 |  |  |  |
| *Peptostreptococcaceae* | 0.026(-0.186, 0.238) | 0.812 | 0.973 | 0.098 | -0.008(-0.371, 0.355) | 0.967 | -0.010(-0.20, 0.180) | 0.930 | -0.136(-1.328, 1.056) | 0.860 | 0.829 |  |  |  |
| *Actinobacteria^1^* | -0.02(-0.157, 0.117) | 0.771 | 0.973 | 0.991 | -0.022(-0.826, 0.782) | 0.958 | -0.025(-0.205, 0.155) | 0.808 | -0.053(-0.621, 0.515) | 0.885 | 0.926 |  |  |  |
| *Bifidobacteriaceae* | -0.035(-0.178, 0.108) | 0.636 | 0.973 | 0.717 | -0.055(-0.382, 0.272) | 0.741 | -0.063(-0.261, 0.135) | 0.595 | -0.160(-0.756, 0.436) | 0.691 | 0.743 |  |  |  |
| *Oxalobacteraceae* | 0.058(-0.187, 0.303) | 0.644 | 0.973 | 0.607 | 0.047(-0.345, 0.439) | 0.812 | 0.157(-0.139, 0.453) | 0.407 | 0.535(-0.474, 1.544) | 0.488 | 0.515 |  |  |  |
| *Streptococcaceae* | 0.021(-0.116, 0.158) | 0.762 | 0.973 | 0.928 | 0.023(-0.197, 0.243) | 0.835 | 0.026(-0.139, 0.191) | 0.791 | 0.047(-0.517, 0.611) | 0.897 | 0.942 |  |  |  |
| *Eubacterium coprostanoligenes group* | -0.012(-0.224, 0.200) | 0.910 | 0.973 | 0.079 | 0.011(-0.567, 0.589) | 0.971 | 0.115(-0.067, 0.297) | 0.345 | 0.581(0.03, 1.132) | 0.287 | 0.275 |  |  |  |
| *Ruminococcus torques group* | 0.007(-0.124, 0.138) | 0.911 | 0.973 | 0.956 | 0.009(-0.363, 0.381) | 0.961 | 0.019(-0.15, 0.188) | 0.845 | 0.085(-0.46, 0.63) | 0.811 | 0.822 |  |  |  |
| *Allisonella* | 0.066(-0.244, 0.376) | 0.675 | 0.973 | 0.669 | 0.077(-4.127, 4.281) | 0.971 | -0.053(-0.439, 0.333) | 0.813 | -0.498(-1.78, 0.784) | 0.585 | 0.537 |  |  |  |
| *Enterorhabdus* | -0.019(-0.221, 0.183) | 0.855 | 0.973 | 0.410 | -0.088(-5.221, 5.045) | 0.973 | -0.09(-0.347, 0.167) | 0.563 | -0.411(-1.248, 0.426) | 0.512 | 0.518 |  |  |  |
| *Erysipelatoclostridium* | -0.034(-0.212, 0.144) | 0.710 | 0.973 | 0.812 | -0.061(-0.33, 0.208) | 0.657 | -0.064(-0.299, 0.171) | 0.645 | -0.207(-0.942, 0.528) | 0.679 | 0.717 |  |  |  |
| *Faecalibacterium* | -0.077(-0.208, 0.054) | 0.251 | 0.973 | 0.398 | -0.088(-0.656, 0.48) | 0.762 | -0.111(-0.303, 0.081) | 0.376 | -0.232(-0.894, 0.43) | 0.617 | 0.718 |  |  |  |
| *Intestinibacter* | 0.034(-0.154, 0.222) | 0.720 | 0.973 | 0.243 | -0.015(-0.323, 0.293) | 0.922 | -0.034(-0.236, 0.168) | 0.773 | -0.453(-1.108, 0.202) | 0.404 | 0.373 |  |  |  |
| *Oxalobacter* | 0.054(-0.281, 0.389) | 0.752 | 0.973 | 0.183 | 0.031(-0.988, 1.05) | 0.953 | 0.257(-0.104, 0.618) | 0.298 | 1.003(-0.061, 2.067) | 0.316 | 0.323 |  |  |  |
| *Peptococcus* | -0.071(-0.304, 0.162) | 0.554 | 0.973 | 0.511 | -0.007(-1.042, 1.028) | 0.989 | 0.001(-0.275, 0.277) | 0.997 | 0.269(-0.695, 1.233) | 0.682 | 0.607 |  |  |  |
| *Peptococcus* | -0.071(-0.304, 0.163) | 0.554 | 0.973 | 0.360 | -0.007(-0.635, 0.621) | 0.511 | 0.001(-0.299, 0.300) | 0.997 | 0.269(-0.696, 1.233) | 0.682 | 0.607 |  |  |  |
| *Romboutsia* | -0.027(-0.305, 0.251) | 0.849 | 0.973 | 0.028 | -0.018(-0.475, 0.439) | 0.938 | -0.021(-0.231, 0.189) | 0.862 | -0.109(-1.728, 1.51) | 0.916 | 0.935 |  |  |  |
| *RuminococcaceaeUCG013* | -0.006(-0.141, 0.129) | 0.926 | 0.973 | 0.638 | -0.002(-0.335, 0.331) | 0.992 | -0.061(-0.237, 0.115) | 0.566 | -0.269(-0.83, 0.292) | 0.519 | 0.518 |  |  |  |
| *RuminococcaceaeUCG009* | -0.046(-0.248, 0.156) | 0.660 | 0.973 | 0.690 | -0.041(-0.392, 0.31) | 0.818 | 0.026(-0.237, 0.289) | 0.862 | 0.297(-0.54, 1.134) | 0.614 | 0.561 |  |  |  |
| *Ruminococcus1* | 0.016(-0.233, 0.265) | 0.901 | 0.973 | 0.035 | -0.002(-0.402, 0.398) | 0.993 | -0.136(-0.342, 0.07) | 0.327 | -0.701(-1.265, -0.137) | 0.248 | 0.236 |  |  |  |
| *Streptococcus* | 0.037(-0.100, 0.174) | 0.601 | 0.973 | 0.656 | 0.045(-0.186, 0.276) | 0.703 | 0.054(-0.122, 0.23) | 0.610 | 0.120(-0.448, 0.688) | 0.749 | 0.816 |  |  |  |
| *Tyzzerella3* | 0.225(-0.143, 0.593) | 0.231 | 0.973 | 0.094 | 0.155(-1.550, 1.860) | 0.859 | 0.176(-0.153, 0.505) | 0.405 | 0.341(-1.793, 2.475) | 0.807 | 0.930 |  |  |  |
| *Gastranaerophilales* | 0.023(-0.206, 0.252) | 0.841 | 0.973 | 0.977 | 0.034(-0.446, 0.514) | 0.890 | 0.037(-0.232, 0.306) | 0.812 | 0.104(-0.843, 1.051) | 0.865 | 0.892 |  |  |  |
| *Actinobacteria^2^* | 0.012(-0.119, 0.143) | 0.856 | 0.973 | 0.638 | 0.025(-0.189, 0.239) | 0.821 | 0.044(-0.125, 0.213) | 0.662 | 0.226(-0.315, 0.767) | 0.564 | 0.572 |  |  |  |
| Colorectal neoplasia (CRC + adenoma) | |  |  |  |  |  |  |  |  |  |  |  |  |  |
| *Gammaproteobacteria* | 0.017(0.002, 0.032) | 0.023 | 0.375 | 0.149 | 0.028(0.009, 0.047) | 0.005 | 0.020(0.004, 0.036) | 0.014 | 0.024(0.006, 0.042) | 0.013 | 0.122 | 0.017(0.002, 0.032) | 0.025 | 0.119 |
| *Lactobacillales* | 0.001(-0.012, 0.015) | 0.872 | 0.973 | 0.362 | 0.003(-0.017, 0.023) | 0.800 | 0.001(-0.015, 0.017) | 0.922 | 0.003(-0.013, 0.019) | 0.735 | 0.715 | -0.003(-0.081, 0.076) | 0.949 | 0.128 |
| *Enterobacteriaceae* | 0.013(-0.001, 0.028) | 0.077 | 0.642 | 0.424 | 0.012(-0.01, 0.034) | 0.275 | 0.013(-0.003, 0.029) | 0.125 | 0.016(-0.002, 0.034) | 0.081 | 0.578 | 0.042(-0.044, 0.127) | 0.343 | 0.276 |
| *Porphyromonadaceae* | -0.001(-0.014, 0.011) | 0.818 | 0.973 | 0.746 | -0.008(-0.027, 0.011) | 0.433 | -0.003(-0.017, 0.011) | 0.684 | 0.001(-0.015, 0.017) | 0.925 | 0.618 | 0.076(0.008, 0.143) | 0.031 | 0.236 |
| *Fusobacteriaceae* | 0.003(-0.011, 0.017) | 0.653 | 0.973 | 0.623 | 0.008(-0.012, 0.028) | 0.429 | 0.012(-0.021, 0.045) | 0.497 | -0.011(-0.048, 0.026) | 0.550 | 0.406 | -0.019(-0.051, 0.012) | 0.237 | 0.780 |
| *Actinomyces* | 0.012(-0.008, 0.031) | 0.241 | 0.973 | 0.768 | 0.019(-0.009, 0.047) | 0.176 | 0.009(-0.015, 0.033) | 0.436 | 0.009(-0.015, 0.033) | 0.436 | 0.748 | -0.045(-0.149, 0.059) | 0.396 | 0.801 |
| *Bifidobacterium* | 0.001(-0.014, 0.015) | 0.934 | 0.973 | 0.285 | -0.003(-0.025, 0.019) | 0.805 | 0.035(-0.012, 0.082) | 0.156 | -0.002(-0.02, 0.016) | 0.806 | 0.581 | 0.018(-0.065, 0.101) | 0.675 | 0.219 |
| *Roseburia* | -0.008(-0.024, 0.007) | 0.283 | 0.973 | 0.001 | -0.001(-0.019, 0.017) | 0.941 | -0.005(-0.021, 0.011) | 0.532 | -0.001(-0.019, 0.017) | 0.957 | 0.143 | -0.034(-0.124, 0.055) | 0.453 | 0.001 |
| *Peptostreptococcaceae* | 0.001(-0.016, 0.016) | 0.999 | 0.999 | 0.252 | 0.002(-0.018, 0.022) | 0.826 | 0.002(-0.014, 0.018) | 0.781 | 0.003(-0.013, 0.019) | 0.757 | 0.419 | 0.001(-0.016, 0.016) | 0.983 | 0.271 |
| *Actinobacteria^1^* | 0.002(-0.012, 0.016) | 0.787 | 0.973 | 0.512 | 0.003(-0.017, 0.023) | 0.783 | 0.005(-0.011, 0.021) | 0.513 | 0.002(-0.014, 0.018) | 0.836 | 0.914 | 0.002(-0.012, 0.016) | 0.818 | 0.519 |
| *Bifidobacteriaceae* | 0.001(-0.015, 0.017) | 0.876 | 0.973 | 0.292 | -0.002(-0.024, 0.02) | 0.831 | 0.001(-0.016, 0.016) | 0.967 | 0.001(-0.018, 0.018) | 0.955 | 0.602 | 0.001(-0.015, 0.017) | 0.868 | 0.256 |
| *Oxalobacteraceae* | -0.011(-0.038, 0.016) | 0.446 | 0.973 | 0.087 | -0.021(-0.056, 0.014) | 0.253 | -0.011(-0.038, 0.016) | 0.425 | -0.012(-0.041, 0.017) | 0.414 | 0.753 | -0.012(-0.039, 0.015) | 0.419 | 0.042 |
| *Streptococcaceae* | -0.004(-0.018, 0.01) | 0.552 | 0.973 | 0.543 | -0.001(-0.021, 0.019) | 0.918 | -0.005(-0.021, 0.011) | 0.532 | -0.002(-0.018, 0.014) | 0.820 | 0.403 | -0.004(-0.018, 0.01) | 0.629 | 0.414 |
| *Eubacterium coprostanoligenes group* | 0.005(-0.009, 0.019) | 0.510 | 0.973 | 0.988 | 0.009(-0.011, 0.029) | 0.360 | 0.006(-0.008, 0.02) | 0.408 | 0.002(-0.014, 0.018) | 0.742 | 0.470 | 0.004(-0.008, 0.016) | 0.477 | 0.996 |
| *Ruminococcus torques group* | 0.003(-0.013, 0.019) | 0.723 | 0.973 | 0.010 | 0.005(-0.017, 0.027) | 0.631 | 0.006(-0.01, 0.022) | 0.415 | 0.006(-0.012, 0.024) | 0.528 | 0.425 | 0.003(-0.013, 0.019) | 0.702 | 0.017 |
| *Allisonella* | -0.007(-0.054, 0.04) | 0.785 | 0.973 | 0.783 | 0.025(-0.044, 0.094) | 0.472 | -0.009(-0.06, 0.042) | 0.729 | -0.009(-0.06, 0.042) | 0.722 | 0.796 | -0.008(-0.051, 0.035) | 0.730 | 0.751 |
| *Enterorhabdus* | 0.005(-0.017, 0.027) | 0.627 | 0.973 | 0.194 | 0.005(-0.028, 0.038) | 0.759 | 0.005(-0.019, 0.029) | 0.678 | 0.013(-0.011, 0.037) | 0.274 | 0.095 | 0.005(-0.017, 0.027) | 0.644 | 0.269 |
| *Erysipelatoclostridium* | -0.003(-0.023, 0.017) | 0.754 | 0.973 | 0.349 | -0.003(-0.028, 0.022) | 0.819 | -0.003(-0.023, 0.017) | 0.752 | -0.003(-0.023, 0.017) | 0.758 | 0.954 | -0.003(-0.021, 0.015) | 0.763 | 0.456 |
| *Faecalibacterium* | -0.003(-0.017, 0.011) | 0.690 | 0.973 | 0.424 | 0.001(-0.020, 0.020) | 0.964 | -0.002(-0.016, 0.012) | 0.734 | -0.004(-0.02, 0.012) | 0.591 | 0.654 | -0.003(-0.017, 0.011) | 0.668 | 0.405 |
| *Intestinibacter* | -0.005(-0.023, 0.013) | 0.521 | 0.973 | 0.303 | 0.001(-0.024, 0.026) | 0.920 | -0.006(-0.024, 0.012) | 0.525 | -0.004(-0.022, 0.014) | 0.647 | 0.732 | -0.006(-0.022, 0.01) | 0.486 | 0.297 |
| *Oxalobacter* | 0.001(-0.026, 0.028) | 0.968 | 0.992 | 0.236 | -0.018(-0.057, 0.021) | 0.376 | -0.001(-0.028, 0.026) | 0.925 | -0.001(-0.032, 0.03) | 0.959 | 0.817 | -0.001(-0.03, 0.028) | 0.968 | 0.142 |
| *Peptococcus* | -0.006(-0.031, 0.019) | 0.655 | 0.973 | 0.212 | -0.010(-0.043, 0.023) | 0.567 | -0.009(-0.036, 0.018) | 0.515 | -0.016(-0.043, 0.011) | 0.260 | 0.065 | -0.006(-0.031, 0.019) | 0.632 | 0.150 |
| *Peptococcus* | -0.005(-0.028, 0.019) | 0.705 | 0.973 | 0.211 | -0.013(-0.047, 0.021) | 0.464 | -0.007(-0.031, 0.017) | 0.574 | -0.015(-0.044, 0.014) | 0.297 | 0.197 | 0.070 (-0.040, 0.235) | 0.169 | 0.107 |
| *Romboutsia* | -0.007(-0.023, 0.009) | 0.357 | 0.973 | 0.624 | 0.005(-0.017, 0.027) | 0.659 | -0.004(-0.022, 0.014) | 0.650 | -0.005(-0.021, 0.011) | 0.522 | 0.587 | -0.007(-0.023, 0.009) | 0.355 | 0.621 |
| *RuminococcaceaeUCG013* | -0.004(-0.002, 0.012) | 0.578 | 0.973 | 0.075 | 0.001(-0.022, 0.022) | 0.998 | -0.002(-0.018, 0.014) | 0.843 | 0.001(-0.015, 0.017) | 0.861 | 0.072 | -0.005(-0.021, 0.011) | 0.494 | 0.074 |
| *RuminococcaceaeUCG009* | -0.023(-0.045, -0.001) | 0.043 | 0.538 | 0.156 | -0.022(-0.055, 0.011) | 0.191 | -0.020(-0.045, 0.005) | 0.112 | -0.028(-0.052, -0.004) | 0.027 | 0.331 | -0.022(-0.046, 0.002) | 0.063 | 0.132 |
| *Ruminococcus1* | -0.003(-0.019, 0.013) | 0.669 | 0.973 | 0.126 | 0.001(-0.024, 0.024) | 0.996 | -0.002(-0.018, 0.014) | 0.832 | -0.004(-0.02, 0.012) | 0.649 | 0.869 | -0.004(-0.02, 0.012) | 0.601 | 0.107 |
| *Streptococcus* | -0.004(-0.018, 0.01) | 0.579 | 0.973 | 0.576 | -0.002(-0.022, 0.018) | 0.878 | -0.004(-0.02, 0.012) | 0.576 | -0.002(-0.018, 0.014) | 0.793 | 0.516 | -0.003(-0.017, 0.011) | 0.646 | 0.513 |
| *Tyzzerella3* | -0.019(-0.044, 0.006) | 0.134 | 0.815 | 0.404 | -0.033(-0.074, 0.008) | 0.112 | -0.014(-0.043, 0.015) | 0.382 | -0.025(-0.052, 0.002) | 0.085 | 0.350 | -0.020(-0.045, 0.005) | 0.137 | 0.180 |
| *Gastranaerophilales* | 0.009(-0.015, 0.033) | 0.432 | 0.973 | 0.667 | 0.004(-0.029, 0.037) | 0.797 | 0.002(-0.025, 0.029) | 0.904 | 0.015(-0.01, 0.04) | 0.258 | 0.284 | 0.009(-0.013, 0.031) | 0.448 | 0.701 |
| *Actinobacteria^2^* | 0.005(-0.009, 0.019) | 0.436 | 0.973 | 0.828 | -0.001(-0.021, 0.019) | 0.904 | 0.006(-0.01, 0.022) | 0.423 | 0.006(-0.008, 0.02) | 0.455 | 0.936 | 0.005(-0.007, 0.017) | 0.441 | 0.812 |

MR, mendelian randomization; β, effect estimates for exposure to outcome, which refers to the Wald ratio combined in a fixed-effect meta-analysis after weighing each ratio estimate and reflects the log-transformed abundance changes in the abundance of gut microbiota for per one-unit increase in the log odds ratio of colorectal neoplasia; FDR, false discovery rate; IVW, inverse variance weighted; MR-PRESSO, MR pleiotropy residual sum and outlier test.

^1^ class *Actinobacteria*

**^2^** phylum *Actinobacteria*
